# Environmental Response and Genomic Regions Correlated with Rice Root Growth and Yield under Drought in the OryzaSNP Panel across Multiple Study Systems

**DOI:** 10.1371/journal.pone.0124127

**Published:** 2015-04-24

**Authors:** Len J. Wade, Violeta Bartolome, Ramil Mauleon, Vivek Deshmuck Vasant, Sumeet Mankar Prabakar, Muthukumar Chelliah, Emi Kameoka, K. Nagendra, K. R. Kamalnath Reddy, C. Mohan Kumar Varma, Kalmeshwar Gouda Patil, Roshi Shrestha, Zaniab Al-Shugeairy, Faez Al-Ogaidi, Mayuri Munasinghe, Veeresh Gowda, Mande Semon, Roel R. Suralta, Vinay Shenoy, Vincent Vadez, Rachid Serraj, H. E. Shashidhar, Akira Yamauchi, Ranganathan Chandra Babu, Adam Price, Kenneth L. McNally, Amelia Henry

**Affiliations:** 1 Charles Sturt University, Graham Centre for Agricultural Innovation, Locked Bag 588, Wagga Wagga, New South Wales, 2678, Australia; 2 International Rice Research Institute (IRRI), DAPO Box 7777, Metro Manila, 1301, Philippines; 3 Center for Plant Molecular Biology and Biotechnology, Tamil Nadu Agricultural University, Coimbatore, 641 003, India; 4 Graduate School of Bioagricultural Sciences, Nagoya University Chikusa, Nagoya, 464–8601, Japan; 5 Barwale Foundation, Barwale Chambers, Street No.10, Himayat nagar, Hyderabad, 500029, India; 6 Institute of Biological and Environmental Sciences, University of Aberdeen, Aberdeen, AB24 3UU, United Kingdom; 7 Africa Rice Center, c/o IITA, PMB 5320 Oyo Road, Ibadan, Nigeria; 8 Philippine Rice Research Institute, Maligaya, Science City of Muñoz, 3119, Nueva Ecija, Philippines; 9 International Crops Research Institute for the Semi-Arid-Tropics (ICRISAT), Patancheru, Telangana, 502324, India; 10 College of Agriculture, University of Agricultural Sciences—Gandhi Krishi Vignana Kendra (GKVK), Bangalore, 560065, India; National Institute of Plant Genome Research (NIPGR), INDIA

## Abstract

The rapid progress in rice genotyping must be matched by advances in phenotyping. A better understanding of genetic variation in rice for drought response, root traits, and practical methods for studying them are needed. In this study, the OryzaSNP set (20 diverse genotypes that have been genotyped for SNP markers) was phenotyped in a range of field and container studies to study the diversity of rice root growth and response to drought. Of the root traits measured across more than 20 root experiments, root dry weight showed the most stable genotypic performance across studies. The environment (E) component had the strongest effect on yield and root traits. We identified genomic regions correlated with root dry weight, percent deep roots, maximum root depth, and grain yield based on a correlation analysis with the phenotypes and aus, indica, or japonica introgression regions using the SNP data. Two genomic regions were identified as hot spots in which root traits and grain yield were co-located; on chromosome 1 (39.7–40.7 Mb) and on chromosome 8 (20.3–21.9 Mb). Across experiments, the soil type/ growth medium showed more correlations with plant growth than the container dimensions. Although the correlations among studies and genetic co-location of root traits from a range of study systems points to their potential utility to represent responses in field studies, the best correlations were observed when the two setups had some similar properties. Due to the co-location of the identified genomic regions (from introgression block analysis) with QTL for a number of previously reported root and drought traits, these regions are good candidates for detailed characterization to contribute to understanding rice improvement for response to drought. This study also highlights the utility of characterizing a small set of 20 genotypes for root growth, drought response, and related genomic regions.

## Introduction

Strategies to address the urgent need for improved rice productivity under drought stress, which affects 23 million hectares in Asia alone [1], will likely be most effective with the collective inputs from multiple research backgrounds [2]. The rice research community is deploying various approaches to understand and improve rice response to drought, including direct selection for grain yield under drought and selecting for traits, such as deep root growth [3]. Considerable genetic variability exists among rice germplasm for grain yield under drought, as well as for traits associated with root growth and drought response [4, 5, 6, 7]. In this study, we focused on a range of phenotyping systems for deep root growth that were collectively analyzed on a panel of diverse germplasm with known SNP haplotypes and pre-defined patterns of introgression blocks, enabling genomic regions linked with deep root growth and grain yield under drought to be correlated, with an aim to better understand these traits and phenotyping systems in order to better improve the response of rice to drought stress.

Given the general tendency of rice to grow roots mostly in shallow zones of the soil profile, deep root growth has long been considered an advantageous trait for improving the performance of rice under drought stress [6, 8, 9]. Significant efforts have been invested in this area of research, resulting in the identification of many quantitative trait loci (QTLs) for root traits related to deep root growth (as reviewed by [10, 11, 12]). In most cases, however, direct correlations or causative relationships with enhanced grain yield under drought stress have not been established with deep-root related traits either individually or collectively. Since it is the grain yield that matters for a farmer, whose crop has been challenged by drought and who has to make the best use of available water, these results point to the necessity of concurrently measuring grain yield while evaluating the trait(s) thought to improve the response to drought stress.

In addition to the uncertainty about the link between certain physiological traits and grain yield, a high degree of environmental variation that is typical to rainfed rice environments [13, 14, 15] may obscure the link between drought response traits and grain yield. Furthermore, the types of study systems used to characterize rice roots (including field, lysimeters, pots, cylinders, root boxes, hydroponics), and the use of different soils, growth media, and treatments of wax layers and polyethylene glycol (PEG) may have influenced the conclusions about the important traits or genetic regions in those studies. For those reasons, in this study we have adopted the approach of combining root studies from many environments and study systems along with grain yield data, in order to identify the most robust root trait responses across experiments that are most likely to improve rice yield under stress.

To take advantage of rice genetic diversity and the currently available genomic tools, another goal of this study was to link root traits with genomic regions. This study was conducted using the OryzaSNP panel [16], which is comprised of 20 diverse rice accessions from the aus, indica, and japonica (with aromatic, temperate and tropical types) groups adapted to a range of agro-ecosystems. The OryzaSNP panel has been genotyped with about 160,000 SNP markers and was analyzed for group-specific haplotype blocks indicative of introgressions from one type into another. The OryzaSNP set has already been used for introgression mapping genomic regions (as haplotype blocks) related to shoot traits [17]. Thus, our approaches in this study were to conduct root studies on highly diverse germplasm that has been genetically well-characterized, to consider a range of root study systems, and to evaluate grain yield along with the root traits, leading to the prospect to identify key traits and genomic regions linked to root growth and drought response in rice by introgression block testing.

## Materials and Methods

### Field and container experiments

**Fig 1:**
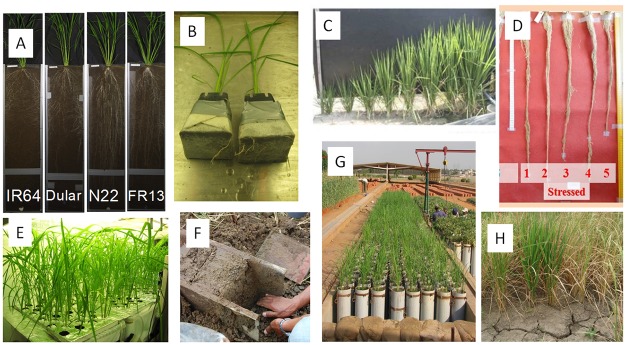
The OryzaSNP germplasm set was phenotyped with a range of root-screening techniques. A) rhizotron (Ab09CR: Univ Aberdeen), B) penetration of nonwoven fabric (Ab09CNWNW: Univ Aberdeen), C) monoliths from line source sprinkler (Na10 and Na11; Nagoya Univ), D) soil-filled cylinders (Ba10C; Barwale Foundation, and CS09C; Charles Sturt Univ), E) hydroponics (Ab09CH: Univ Aberdeen), F) monoliths in the field (IR08FL and IR09dFL; IRRI), G) lysimeters (IR08C; IRRI, IC09C; ICRISAT), and (H) in the field by excavation (TN10F, TN11F; Tamil Nadu Agric. Univ).

The OryzaSNP panel, a set of 20 diverse rice genotypes that have been mapped for 160,000 SNP markers, was used for this study (Table 1). Root experiments were conducted in 10 study systems in different locations and under different conditions, and yield data were collected from 19 field environments (Fig 1; Tables 2 and 3). Detailed protocols for many of these study systems have been published [18].

**Table 1:** The 20 OryzaSNP genotypes used in this study.

Name	Code	Country of origin	Variety type	Subgroup
Aswina	Asw	Bangladesh	ind	Indica
Azucena	Azu	Philippines	trop	Japonica
Cypress	Cyp	USA	trop	Japonica
Dom Sufid	Dom	Iran	aro	Japonica
Dular	Dul	India	aus	Aus
FR13A	FR1	India	aus	Aus
IR 64	IR64	Philippines	ind	indica
LTH	LTH	China	temp	japonica
M 202	M202	USA	temp	japonica
Minghui 63	MH63	China	ind	indica
Moroberekan	Moro	Guinea	trop	japonica
N22	N22	India	aus	Aus
Nipponbare	Nip	Japan	temp	japonica
Pokkali	Pok	India	ind	indica
Rayada	Ray	Bangladesh	aus	aus
Sadu Cho	Scho	Korea	ind	indica
San Huang Zhan 2	Shz2	China	ind	indica
Swarna	Swa	India	ind	indica
Tainung 67	TNG	Taiwan	temp	japonica
Zhenshan 97B	Zhe	China	ind	indica

**Table 2:** Summary of all experiments analyzed in this study.

Expt		Location	Year/Season	Environment	Description	Treatment	Root sampling (DAS)	Grain yield	RDW	%DR	MRL
**Field studies**											
1	Af12FC	Ibadan, Nigeria	2012 Jan-Jul	field	transplanted lowland	well-watered control (aerobic)		√			
2	Af12FS	Ibadan, Nigeria	2012 Jan-Jul	field	transplanted lowland	drought stress (aerobic)		√			
3	Ba10FC	Maharajpet, Andhra Pradesh, India	2010 Jun-Nov (wet season)	field	transplanted lowland	flooded control		√			
4	Ba10FS	Maharajpet, Andhra Pradesh, India	2010 Jun-Nov (wet season)	field	transplanted lowland	drought stress (initially flooded)		√			
5	IR08FLC	Los Baños, Laguna, Philippines	2008 Jan-Apr (dry season)	field	transplanted lowland	flooded control	72	√	√	√	
6	IR08FLS	Los Baños, Laguna, Philippines	2008 Jan-Apr (dry season)	field	transplanted lowland	drought stress (initially flooded)	72	√	√	√	
7	IR09dFLC	Los Baños, Laguna, Philippines	2009 Jan-Apr (dry season)	field	transplanted lowland	flooded control		√			
8	IR09dFLS	Los Baños, Laguna, Philippines	2009 Jan-Apr (dry season)	field	transplanted lowland	drought stress (initially flooded)	92	√	√		
9	IR09wFLC	Los Baños, Laguna, Philippines	2009 Jul-Oct (wet season)	field	transplanted lowland	flooded control		√			
10	IR09wFLS	Los Baños, Laguna, Philippines	2009 Jul-Oct (wet season)	field	transplanted lowland	drought stress (initially flooded)		√			
11	IR10FLC	Los Baños, Laguna, Philippines	2010 Jan-Apr (dry season)	field	transplanted lowland	flooded control		√			
12	IR10FLS	Los Baños, Laguna, Philippines	2010 Jan-Apr (dry season)	field	transplanted lowland	drought stress (initially flooded)		√			
13	IR10UFC	Los Baños, Laguna, Philippines	2010 Jan-Apr (dry season)	field	direct-seeded upland	well-watered control (aerobic)		√			
14	IR12FLS	Los Baños, Laguna, Philippines	2012 Jul-Oct (wet season)	field	transplanted lowland	flooded control		√			
15	IR12FLC	Los Baños, Laguna, Philippines	2012 Jul-Oct (wet season)	field	transplanted lowland	drought stress (initially flooded)		√			
16	Na10FINT	Nagoya, Japan	2010 (May-Oct)	fixed rainout shelter	transplanted, line source	int. drought stress (aerobic)	134–150		√		√
17	Na10FWD	Nagoya, Japan	2010 (May-Oct)	fixed rainout shelter	transplanted, line source	severe drought stress (aerobic)	134–150		√		√
18	Na10FWW	Nagoya, Japan	2010 (May-Oct)	fixed rainout shelter	transplanted, line source	well-watered control (aerobic)	134–150		√		√
19	Na11FINT	Nagoya, Japan	2011 (May-Sep)	fixed rainout shelter	transplanted, line source	int. drought stress (aerobic)	90–113		√		√
20	Na11FWD	Nagoya, Japan	2011 (May-Sep)	fixed rainout shelter	transplanted, line source	severe drought stress (aerobic)	90–113		√		√
21	Na11FWW	Nagoya, Japan	2011 (May-Sep)	fixed rainout shelter	transplanted, line source	well-watered control (aerobic)	90–113		√		√
22	TN10FC	Paramakudi, Tamil Nadu, India	2010–2011 Sep-Jan (wet season)	field	lowland (flooded)	flooded control	115	√	√		√
23	TN10FS	Paramakudi, Tamil Nadu, India	2010–2011 Sep-Jan (wet season)	field	lowland (initially flooded)	drought stress (initially flooded)	113	√	√		√
24	TN11FC	Paramakudi, Tamil Nadu, India	2011–2012 Sep-Jan (wet season)	field	lowland (flooded)	flooded control	125	√	√	√	√
25	TN11FS	Paramakudi, Tamil Nadu, India	2011–2012 Sep-Jan (wet season)	field	lowland (initially flooded)	drought stress (initially flooded)	130	√	√	√	√
**Container studies**											
1	Ab09CH	Aberdeen, UK	2009 Mar	growth chamber	38-L (58 cm x 38.5 cm x 26 cm) aerated hydroponic tank, 36 plants per tank	n/a	35		√	√	√
2	Ab09CNW	Aberdeen, UK	2009 Mar-Sep	growth chamber	0.5-L (8 x 8 cm at top, 5.5 x 5.5 cm at bottom, 12.3 cm deep) soil-filled pot, covered at the bottom with non-woven fabric (aerobic)	n/a	38		√	√	
3	Ab09CR	Aberdeen, UK	2009–2010 Jun-Jan	greenhouse	5.4-L (30 cm x 1.5 cm x 120 cm) soil-filled rhizotron (aerobic)	n/a	42		√		√
4	Ba10CC	Maharajpet, Andhra Pradesh, India	2010 Jul-Oct	outdoor	60-L (25 cm x 120 cm) soil-filled cylinders (aerobic)	well-watered control	100		√		√
5	Ba10CS	Maharajpet, Andhra Pradesh, India	2010 Jul-Oct			drought stress	100		√		√
6	CS09CC	Wagga Wagga, NSW, Australia	2009 Oct–Nov	greenhouse	15-L (20 cm x 50 cm) soil-filled cylinders (all initially flooded)	well-watered control	42		√	√	
7	CS10CS1	Wagga Wagga, NSW, Australia	2010 Feb-Mar			drought stress (intermediate)	42		√	√	
8	CS10CS2	Wagga Wagga, NSW, Australia	2010 Apr-May			drought stress (severe)	42		√	√	
9	IC09CC	Patencheru, Andhra Pradesh, India	2009 Jan-Apr (dry season)	outdoor rainout shelter	37-L (20 cm x 120 cm) soil-filled cylinders (all initially flooded)	well-watered control	96		√		
10	IC09CS	Patencheru, Andhra Pradesh, India	2009 Jan-Apr (dry season)			drought stress	96		√		
11	IR08CC	Los Baños, Laguna, Philippines	2008 Sep-Dec (wet season)	greenhouse	29-L (19 cm x 105 cm) soil-filled cylinders (all initially flooded)	well-watered control	74		√	√	√
12	IR08CS	Los Baños, Laguna, Philippines	2008 Sep-Dec (wet season)			drought stress	74		√	√	√

Experiment names include the first two letters of the institute where the experiment was conducted, the year in which the experiment was conducted, letters indicating the type of study (F (field) or C (container)), the season (w (wet season) or d (dry season)), system (L (lowland), U (upland), H (hydroponics), NW (non-woven fabric), or R (rhizotron)), and a letter for the treatment (C (control), S (stress), INT (intermediate stress), WD (water deficit), or WW (well-watered). DAS = days after sowing.

**Table 3:** Environmental conditions across all field and container studies.

Expt		Latitude, Longitude	Soil type	% Sand	% Silt	% Clay	pH	Bulk density (g cm^-3^)	Fertilizer applied (kg NPK ha^-1^)	Irrigation applied	Rain (mm)	Evap. (mm)	Ave. temp. (°C)	Mean solar rad. (MJ m^–2^ d^–1^)	Ave.RH (%)
**Field studies**															
1	Af12FC	7° 29'N, 3° 54' E	Sandy clay silt	69	12	20	5.3	-	200 kg ha^-1^ 15:15:15, and 2 urea topdressings	Sprinkler	-	-	-	-	-
2	Af12FS			-	-	-	-	-		Rainfed	-	-	-	-	-
3	Ba10FC	17° 24'N, 78° 13' E	Black clay	34.4	12	53.6	7.94	1.23	100:60:40 basal; and 2 urea topdressings (15 kg ha^-1^)	Flooded	910	652	27.0	-	-
4	Ba10FS									Drained 6 WAT	910	652	27.0	-	-
5	IR08FLC	14° 10'N, 121° 15' E	Aquandic Epiaquall	-	-	-	-	-	40:40:40 before planting, then 40 kg N ha^-1^ 3–4 weeks after planting	Flooded	385	581	27.0	15.6	84.1
6	IR08FLS			20	40	40	5.9			Drained 4 WAT	385	581	27.0	15.6	84.1
7	IR09dFLC		Isohyperthermic Typic Hapludalf	-	-	-	-	-		Flooded	721	589	26.6	15.4	86.8
8	IR09dFLS			26	48	26	5.6	0.83		Drained 4 WAT	721	589	26.6	15.4	86.8
9	IR09wFLC			-	-	-	-	-		Flooded	1406	570	27.8	14.2	89.7
10	IR09wFLS			33	42	25	5.6	1.08		Drained 4 WAT	1406	570	27.8	14.2	89.7
11	IR10FLC			-	-	-	-	-		Flooded	107	733	26.8	17.0	87.1
12	IR10FLS			24	50	26	5.6	1.02		Drained 4 WAT	107	733	26.8	17.0	87.1
13	IR10UFC		Isohyperthermic, Mixed typic Tropaquept	36	40	24		1.244		Sprinkler, to supplement rainfall	107	733	26.8	17.0	87.1
14	IR12FLS		Isohyperthermic Typic Hapludalf	-	-	-	-	-		Drained 4 WAT	1240	503	24.0	14.0	90.0
15	IR12FLC			24	50	26	5.6	1.02		Flooded	1240	503	24.0	14.0	90.0
16	Na10FINT	35° 11' N, 156° 52' E	Sandy loam	72.3	20.9	6.8	5.5	1.3	80:100:75	Line-source sprinkler	na	-	28.7	-	71.6
17	Na10FWD									Line-source sprinkler	na	-	28.7	-	71.6
18	Na10FWW									Line-source sprinkler	na	-	28.7	-	71.6
19	Na11FINT								80:80:80	Line-source sprinkler	na	-	26.9	-	72.3
20	Na11FWD									Line-source sprinkler	na	-	26.9	-	72.3
21	Na11FWW									Line-source sprinkler	na	-	26.9	-	72.3
22	TN10FC	9° 33'N, 78° 35' E	Sandy Clay loam	46.8	20	33.2	7.8	-	50:25:25	Flooded	790	-	28.1	15.5	83.1
23	TN10FS								75:25:38	Rainfed	790	-	28.1	15.5	83.1
24	TN11FC								50:25:25	Flooded	345	-	28.0	15.8	86.3
25	TN11FS								75:25:38	Rainfed	345	-	28.0	15.8	86.3
**Container studies**															
1	Ab09CH	57° 8'N, 2° 5' W	Nutrient solution	na	na	na	5.5	na	na	na	na	na	27.0	15.0	
2	Ab09CNW		John Innes No. 2 (2: 1: 2 loam: sand: peat)	-	-	-	5.1	1.1	na	Aerobic fully watered	na	-	27.0	15.0	-
3	Ab09CR		Sandy loam topsoil	-	-	-	5.4	1.1	na	Aerobic fully watered	na	-	27.5	-	-
4	Ba10CC	17° 24'N, 78° 13' E	3.5: 0.5: 1:1 black soil: sand: compost/manure	13	33	54	7.6	-	Basal equal to 100:60:40 kg ha^-1^; 3 g of urea, 4 g P_2_O_5_ and 2 g K_2_O topdressing at 45 DAS; 3 times 2% (w/v) ZnSO_4_ foliar spray	Aerobic well-watered	657	806	27.0	-	-
5	Ba10CS									Irrigation until 30 DAS, and 7 stress cycles	657	806	27.0	-	-
6	CS09CC	35° 3' S, 147° 21' E	Leeton clay loam	28	12	60	5.84	1.46	0.47 g 40:10:10 per cylinder	Flooded	na		25.0	>15.0	
7	CS10CS1									Drained at 21 DAS	na		25.0	>15.0	
8	CS10CS2									Drained at 14 DAS	na		25.0	>15.0	
9	IC09CC	14° 10'N, 121° 15' E	Mollisol	-	-	-	-	1.28	5.9 g 14:14:14 and 5.8 g urea	Flooded	na	-	28.0	15.5	85.2
10	IC09CS									Drained at 44 DAS	na	-	28.0	15.5	85.2
11	IR08CC	17° 30'N, 78° 16' E	Vertisol	-	-	-	-	1.4	5.72 g N, 3.85 g P_2_O_5_, and 2.17 g K_2_O	Flooded	na	-	28.4	20.0	46.3
12	IR08CS									Drained at 70 DAS	na	-	28.4	20.0	46.3

Experiment names include the first two letters of the institute where the experiment was conducted, the year in which the experiment was conducted, letters indicating the type of study (F (field) or C (container)), the season (w (wet season) or d (dry season)), system (L (lowland), U (upland), H (hydroponics), NW (non-woven fabric), or R (rhizotron)), and a letter for the treatment (C (control), S (stress), INT (intermediate stress), WD (water deficit), or WW (well-watered). WAT = weeks after transplanting. (- information not available; na not applicable.)

The root phenotyping systems in this study included soil-filled cylinders of various sizes, soil-filled rhizotrons, cylinders in which root penetration of a non-woven fabric was assessed, hydroponics, a raised-bed line-source system, and field experiments (Fig 1; Table 2). The hydroponics (Ab09CH), rhizotron (Ab09CR), and non-woven fabric (Ab09CNW) experiments at Univ. of Aberdeen, UK, were described previously [19]. In brief, the rhizotron experiments were conducted in soil-filled containers (approx. 5.4 litre volume) made of two sheets of glass (120 cm tall, 30 cm wide) separated by 1.5 cm and inclined at 15°. Plants were grown in a greenhouse for 6 weeks and root trait measurements were made on the lower face of glass every week. Root mass traits were measured at harvest at 6 weeks. The hydroponics setup consisted of 60-liter storage boxes filled with aerated nutrient solution that was changed weekly. Plants were grown in a controlled environment facility for 5 weeks and root traits were measured weekly. The non-woven fabric pot experiment involved attaching a non-woven fabric over the top of a pot (8 x 8 cm square, 12.3 cm deep), cutting off the bottom of the pot, inverting it, and filling it with approximately 600 g of soil. A plant was sown in each pot and grown in the controlled environment facility for 5 weeks. Root penetration through the fabric was recorded. Four replications were used in the rhizotron and non-woven fabric experiments and three replications were used in the hydroponics setup.

Several types of root experiments in cylinders were conducted as part of this study. At the Barwale Foundation Maharajpet Farm, India (Ba10C), roots were evaluated in cylinders with three replications that were assembled from rolled 1-mm thick acrylic sheets with dimensions of 25 cm diam. x 120 cm height and sampled at 100 days after sowing. Cylinders of 19–20 cm diam. X 105–120 cm height were used in lysimeter studies conducted at IRRI, Philippines (IR08C), and ICRISAT, India (IC09C), and were previously reported [20]. Three cylinder studies (20 cm diam. x 50 cm height; CS09C and CS10CS) were conducted by Charles Sturt University, Australia, in the outdoor growth rooms at the Yanco Agricultural Institute, Leeton (see [21]); experiment CS09CC was a well-watered treatment with three replicates, and experiments CS10CS1 and CS10CS2 were water-deficit treatments with four replicates. Pre-germinated seeds were sown in puddled soil, and following emergence, cylinders were flooded to 20 mm depth starting at 7 days after planting, followed by the water-deficit treatments in experiments CS10CS1 and CS10CS2 (Table 3). Soils were thus anaerobic prior to the water deficit, analogous to those of rainfed lowland conditions.

In the field experiments at Tamil Nadu Agricultural University, India (TN10F and TN11F), rice lines were evaluated for drought response and root traits during the wet seasons of 2010 and 2011 under flooded and rainfed conditions in experimental fields of the University at Agricultural Research Station, Paramakudi, India [22, 23, 24]. The experimental details and root sampling and measurement procedures were given previously [25]. Briefly, seeds were sown in dry soil and irrigated as necessary during the initial 5–6 weeks after seedling emergence, after which the field was completely rainfed until harvest and was kept drained throughout the growing season. The flooded treatment plots were irrigated regularly. Drought and well-watered field experiments, mostly under lowland conditions, for yield and root traits were conducted at IRRI (IR08FL, IR09dFL, IR09wFL, IR10FL, IR12FL, and IR10FU) and some of the seasons analyzed here were previously reported [26]. Several other field experiments were conducted for yield in which root traits were not measured. The field experiment at AfricaRice, Nigeria (Af12F), was conducted under direct-seeding (vegetative-stage drought and well-watered treatments) with 0.8 m^2^ plots in two replicates. A lowland field experiment was conducted under well-watered and drought conditions at the Barwale Foundation Maharajpet Farm, India (Ba10F) in 2010 with 0.8 m^2^ plots with two replications in which drought was imposed 45 days from the day of transplanting and irrigation was given once in 15 days to maintain 3–4 cycles of stress across the season. All field studies were conducted on research stations and no specific permission was required for these activities.

The experiments at Nagoya University (Na10F and Na11F) were conducted in a watertight soil bed with line source sprinkler system under a rain-out shelter. The watertight soil bed was constructed with a frame fitted with an impermeable plastic sheet that was then filled with soil, in order to prevent percolation or lateral seepage of water. Irrigation was applied as water mist from the nozzles of a PVC pipe installed at the center of the bed. The soil depth in the bed was 20 cm and the soil moisture gradient was created perpendicular to the water source (PVC pipe) ranging from -2.3 to -10.5 kPa (WW, well-watered treatment), -10.5 to -39.2 kPa (INT, intermediate drought stress), and -39.2 to -285.6 kPa (WD, severed water deficit). Under such conditions, root traits other than deep root growth that contribute to plant growth may be identified for a specified range of drought intensity. Plants were transplanted 21 days after seeding in the bed and grown until heading. Root sampling was conducted using the round monolith method with diameter of 15 cm and height of 20 cm as described previously [27].

The percentage of deep roots (%DR) was calculated from root dry weight data as the proportion of roots below the depth of 20 cm (IR08, CS10CS, Ab09CH) or 30 cm (IR08C, IC09C) in relation to the root dry weight sampled from all depths, except in the NW experiment where the proportion of roots that had penetrated the non-woven fabric in relation to total root dry weight was used. Unifying measurements across all root experiments were maximum root depth (MRL), root dry weight (RDW), and % deep roots (%DR). Shoot dry biomass was determined around the time of root sampling and could be compared between drought stress and control treatments in experiments Ba10C, CS09C, CS10C, IC09C, IR08C, IR08FL, IR09dFL, Na10F, Na11F, and TN11F. Stress response indices were calculated as % shoot biomass reduction by drought [(shoot biomass_control_—shoot biomass_stress_)/shoot biomass_control_ x 100], % RDW reduction by drought [(RDW_control_—RDW _stress_)/ RDW_control_ x 100], % MRL increase by drought [(MRL_stress_—MRL _control_)/ MRL_control_ x 100], %DR increase by drought [%DR_stress_—%DR_control_], and % grain yield reduction by drought [(grain yield_control_—grain yield_stress_)/grain yield_control_ x 100]. The 20 OryzaSNP genotypes studied showed a range of flowering times, including some that were photoperiod sensitive and some that did not flower in certain seasons. Our strategy was to initiate the drought stress early enough to target reproductive stage in all studies in which grain yield was measured, but in some cases no flowering occurred and grain yield was therefore not determined due to photoperiod sensitivity (i.e. in genotypes Nipponbare and Rayada).

### G x E analysis

Least squares means were calculated for each genotype in each experimental environment by mixed model analysis in SAS 9.3 [28] with blocks designated as random effects and genotypes designated as fixed effects. Least squares means from all experiments were compared by Pearson two-sided correlation using the ‘correlation’ script in R 2.15.2 [29]. Ordination analysis using AMMI (mean polish) and location standardized models were conducted to construct biplots with CropStat 7.2 [30]. The levels of GxE interaction (in which the genotype and environment main effects are removed from the model) and GGE (the genotype main effect and the GxE) were explained by the first two principal components (PCA1 and PCA2) from the AMMI and location standardized analyses, respectively. Resulting biplots were graphed using R 1.15.2 [29]. The groupings of genotypes and environments in the biplots were confirmed by dendrograms, constructed using cluster analysis in R. Analyses included all 20 OryzaSNP genotypes; however, some genotypes were absent from certain experiments due to germination problems and the genotype Rayada was excluded from all but the MRL analyses due to its poor germination in several experiments. Missing values are indicated in S1–S4 Tables. Data from Nagoya University were analyzed separately from other environments due to the smaller number of OryzaSNP genotypes used. Correlations among PCA values and environmental characteristics for each experiment were conducted using Pearson correlation.

### Identifying introgression regions for each trait

SNP genotyping data from the 20 OryzaSNP diversity panel [16] were used to correlate yield and root phenotype data using the approach described previously [17] wherein introgression patterns of indica blocks into aus or japonica, aus into indica or japonica, or japonica into indica or aus are defined taking into account population structure. Introgressions were defined as having SNP patterns more similar to the representative SNP pattern of the group for 100kb windows across the genome. The previously identified aus, indica, and japonica genome introgression blocks were correlated with MRL, RDW, %DR, or yield in each respective experimental environment by linear regression and significance checked by one-way ANOVA. Then, a subset of 191 introgression regions correlated with phenotypes was selected using a significance level cutoff value P <0.001. The cutoff value of P<0.001 was chosen in order to present an adequate number of introgression regions to identify co-location of traits at a limited number of sites within the genome. The degrees of freedom for the genetic correlation (DF = 19 for genotypes) and stringent P-value cutoff (<0.001) justified that the set of 20 OryzaSNP genotypes was sufficient for this analysis. The phenotype/introgression region combinations for each experiment were plotted on the rice chromosome Nipponbare genome map (MSU release 6.1; [31]) using Mapchart v. 2.2 [32]. Chromosome regions in which 5 or more traits aligned were selected, and enrichment of published QTLs related to root traits (Gramene QTL) for each of these filtered segments were determined (see R-scripts).

## Results

### Comparison of study systems

**Fig 2:**
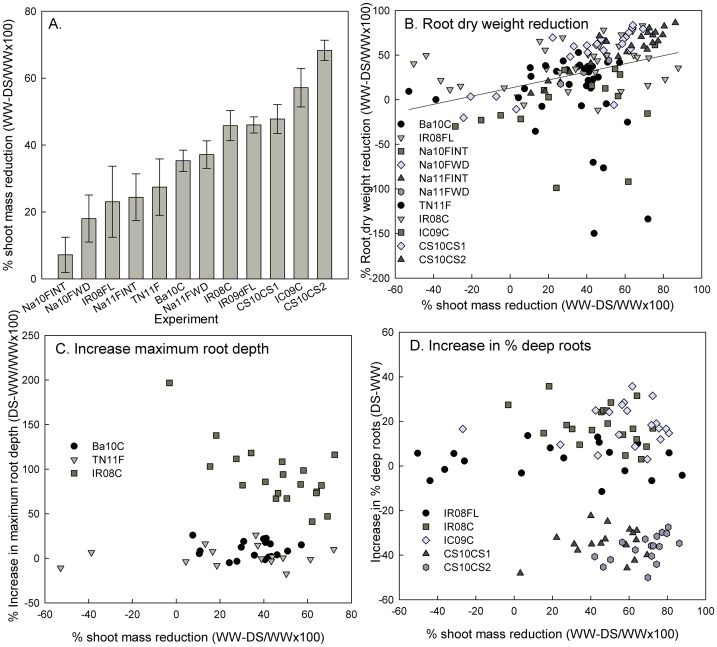
Comparison of genotypic response to drought severity across experiments. A) Shoot mass reduction in the drought treatment compared to the well-watered control. Response of B) root dry weight, C) maximum root depth, and D) % deep roots to increasing drought severity, as indicated by shoot mass reduction. Each data point represents the average difference between DS and WW treatments for one genotype in one experiment. Data previously reported by Henry et al (2011) and Gowda et al (2012) were used to calculate some of the results shown in this figure.

The different growing conditions in each experiment (Tables 2 and 3) resulted in a wide range of drought stress severities achieved across experiments, as evidenced by the yield and root growth responses (S1–S4 Tables) and the average percent reduction in shoot mass of the drought stress treatment (DS) as compared to the well-watered control (WW; Fig 2A).

Three root growth parameters were common to most studies: root dry weight (RDW), maximum root depth (MRL), and % deep roots (%DR; Table 2). Of these, RDW was observed in most studies for both DS and WW treatments and was increasingly reduced with increasing drought severity in almost all experiments and genotypes (Fig 2B). Maximum root depth was not clearly affected by drought stress treatments in field experiment TN11F, but showed a slight increase in cylinder experiment Ba10C and higher increase by drought in cylinder study IR08C, although this trend decreased with increasing drought severity (Fig 2C).The proportion of roots at depth (%DR) was increased by drought stress in all experiments except cylinder studies CS09CS1 and CS09CS2 in which %DR decreased (Fig 2D).

Correlation matrices (S5–S8 Tables, Table 4) indicated a number of corresponding trends in responses among genotypes across the different field and container studies. In general, experiments conducted under well-watered conditions showed more significant correlations with other experiments, and experiments conducted at the same site tended to be significantly correlated (Table 4). Some significant correlations between field and container studies were observed for RDW (S6 Table) and %DR (S7 Table). The non-woven fabric experiment Ab09CNW showed the highest number of significant correlations with other container experiments (Table 4).

**Table 4:** The number of experiments with which the data from each experiment were correlated (p<0.05).

	Significant correlations (# of expts)							
Expt	Grain yield	RDW	MRL	%DR		RDW	MRL	%DR
Field studies					Container studies			
**Well-watered**								
Af12FC	2	-	-	-	Ab09CH	3	4	1
Ba10FC	6	-	-	-	Ab09CNW	8	-	2
IR08FLC	7	6	-	1	Ab09CR	2	3	3
IR09dFLC	7	-	-	-	Ba10CC	3	5	-
IR09wFLC	9	-	-	-	CS09CC	5	-	1
IR10FLC	11	-	-	-	IC09CC	1	-	-
IR10FUC	1	-	-	-	IR08CC	4	3	4
IR12FLC	5	-	-	-				
Na10FWW	-	8	-	-				
Na11FWW	-	7	-	-				
TN10FC	3	1	1	-				
TN11FC	2	2	0	-				
**Drought stress**								
Af12FS	1	-	-	-	Ba10CS	5	3	-
Ba10FS	2	-	-	-	CS10CS1	1	-	1
IR08FLS	1	2	-	1	CS10CS2	2	-	1
IR09dFLS	6	2	-	-	IC09CS	1	-	-
IR09wFLS	8	-	-	-	IR08CS	1	3	1
IR10FLS	9	-	-	-				
IR12FLS	5	-	-	-				
Na10FINT	-	4	-	-				
Na10FWD	-	1	-	-				
Na11FINT	-	2	-	-				
Na11FWD	-	3	-	-				
TN10FS	3	2	1	-				
TN11FS	0	5	1	-				

These numbers are based on Pearson’s two-sided correlation matrix (S1–S5 Tables).

^a^one correlation was negative

### GxE analysis

Across all experiments, the environment (E) component explained the majority of the variation (85–95%) for RDW, and MRL, %DR, and among traits was lowest for yield (60%; Table 5). The genotype x environment (GxE) component was higher for yield (30%) than for all root traits (5–10%), and among root traits the GxE component was highest for RDW (Table 5). The first two principal components explained 56% of the variation for grain yield, 72% of the variation for RDW, 63% of the variation for MRL, and 56% of the variation for %DR. When the AMMI analysis was conducted for yield, RDW, and MRL using only the environments in which both yield and root measurements were conducted, the E effect was still larger for RDW than for grain yield, but E was lower for MRL than for grain yield (S9 Table). Correlations among the first two principle components for each trait with environmental characteristics in each experiment revealed soil texture to be related to RDW, MRL, and %DR, but not to yield (S10 Table).

**Table 5:** AMMI (mean polish) and location standardized model analysis of GxE interactions across all experiments from which yield, root dry weight (RDW), maximum root depth (MRL), and percent deep roots (%DR) were measured.

Variable		Grain Yield	Root dry weight	Maximum root depth	% Deep roots
**Genotype (n)**		19	19	20	19
**Environment (n)**		19	19	10	10
**Df**	**G**	18	18	19	18
	**E**	10	18	9	9
	**GxE**	324	324	171	162
**% variation from Total SS**	**G**	**11.3**	**2.3**	**2.7**	**0.9**
	**E**	**59.3**	**86.2**	**91.7**	**94.6**
	**GxE**	**29.4**	**11.5**	**5.9**	**4.5**
**% variation**	**PC1**	30.3	37.9	36.6	35.8
	**PC2**	25.8	34.5	27.1	20.0
	**PC3**	14.1	8.7	11.7	13.8
	**PC4**	10.3	6.3	9.4	9.2

**Fig 3:**
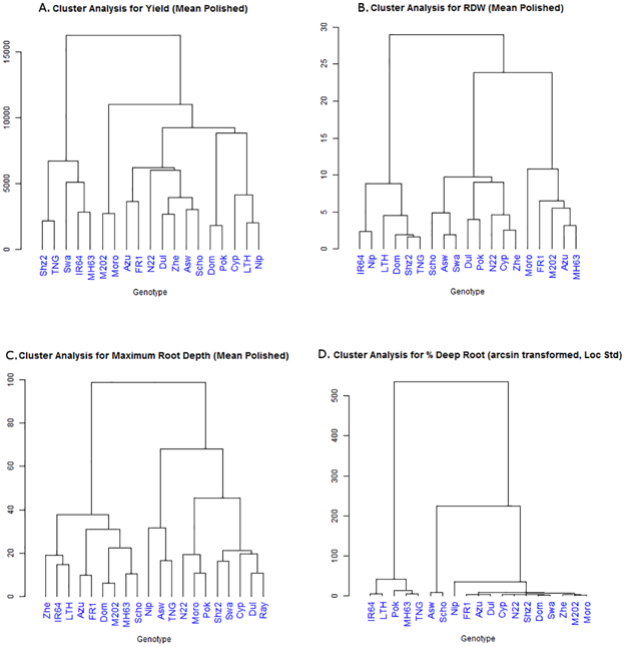
Dendrograms of genotypic groupings. A) grain yield, B) root dry weight, C) maximum root depth, and D) % deep roots. Data previously reported by Henry et al (2011), Gowda et al (2012), and Shrestha et al (2013) were used to calculate some of the results shown in this figure.

**Fig 4:**
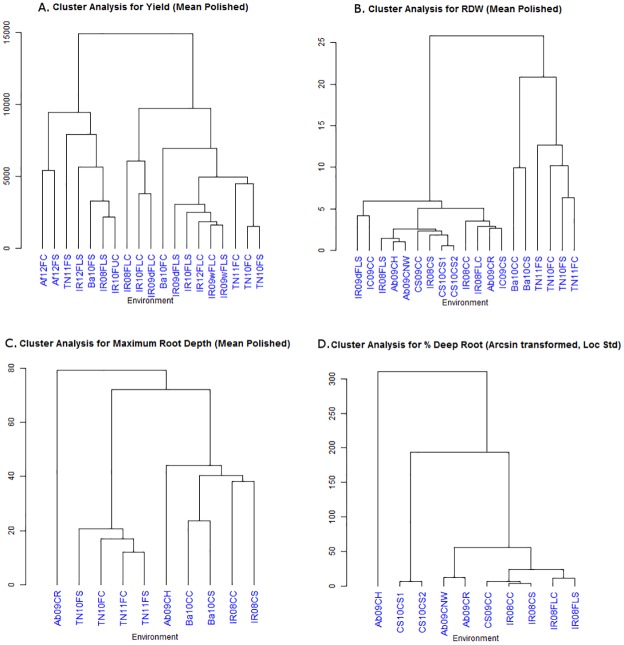
Dendrograms of environmental groupings. A) grain yield, B) root dry weight, C) maximum root depth, and D) % deep roots. Data previously reported by Henry et al (2011), Gowda et al (2012), and Shrestha et al (2013) were used to calculate some of the results shown in this figure.

**Fig 5:**
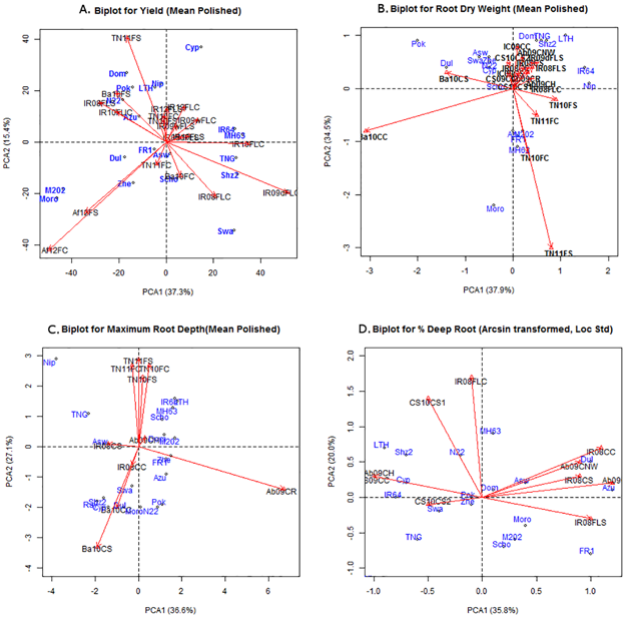
Biplots for A) grain yield, B) root dry weight, C) maximum root depth, and D) % deep roots. Data previously reported by Henry et al (2011), Gowda et al (2012), and Shrestha et al (2013) were used to calculate some of the results shown in this figure.

According to the cluster analysis across all experiments, the genotypes (Table 1) and environments (Table 2) showed different groupings for each trait (Figs 3 and 4). For yield, there appeared to be three distinct groupings of environments in the biplots (Fig 5): those from favorable conditions, those from drought stress treatments (yet including the TNAU well-watered experiment TN11FC), and those for the experiments from AfricaRice (Af12F). For root dry weight, genotypes appeared to group in relation to adaptation, where high-yielding, drought susceptible genotypes including IR64 and Nipponbare were separated from the aus and traditional varieties (Dular, N22, FR13A, Pokkali, etc.; Fig 3). Most environments grouped together for root dry weight, except for Ba10CC, TN11FC, and TN11FS (Fig 5)—all of which exhibited high soil pH (Table 3). Analysis of the subset of seven genotypes evaluated under line-source sprinkler at Nagoya revealed that the root dry weight under stress and well-watered conditions grouped well with other environments in which that treatment was applied (S1 Fig.). Maximum root depth appeared to be most strongly affected by study method, where the rhizotron (Ab09CR) study separated from the large cylinder studies (IR08C, IC09C, Ba10C) and the group of TNAU field studies (TN11F and TN12F; Fig 5). A high degree of diversity was observed among genotypic responses and environments for %DR, as shown by their dispersed distribution in the biplot (Fig 5).

### Chromosome regions correlated with root traits and grain yield

A correlation analysis of aus, indica, and japonica introgression blocks (data from [16]) with MRL, RDW, % deep roots and grain yield identified over 19,000 significant introgression blocks, of which 738 were aus introgressions, 3946 were indica introgressions, and the rest were japonica introgressions. A significance level cutoff value P<0.001 was used to identify a subset of 191 introgression regions most highly correlated with phenotypes, of which two were aus introgressions and 189 were japonica introgressions (S11 Table). Experiments that identified the most significant introgression blocks (and the corresponding number of introgression blocks) were: cylinder experiments Ba10CS (4), Ba10CC (7), IC09CC (10), CS10CS1 (10) IR08CS (19), CS10CS2 (26) IR08CC (28); the rhizotron experiment Ab09CR (3); the non-woven fabric experiment Ab09CNW (3); and the field experiments IR12FLS (1), Af12FC (1), IR08FLC (2), IR12FLC (3), IR10FLC (5), IR08FLS (10), IR10FLS (13), IR09wFLC (21), and IR09wFLS (33). Of the phenotypes correlated with introgression blocks, 1 was for % maximum root depth increase by drought, 7 were for % deep root increase by drought, 17 were for maximum root depth, 37 were for % deep roots, 52 were for root dry weight, and 77 were for grain yield.

**Fig 6:**
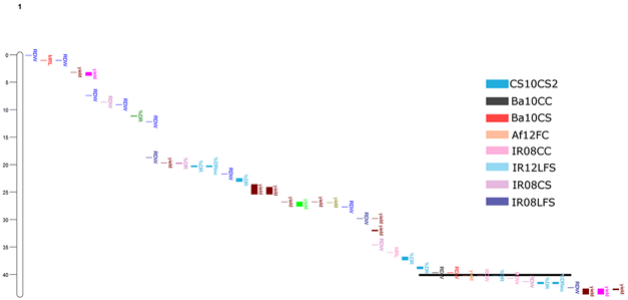
A genomic region on chromosome 1 (39.7–40.7 Mb) was identified as a hot spot in which root dry weight, percent deep roots, and yield aligned. Colors indicate each experiment from which the phenotypic data and introgression regions were correlated. Experiments from which introgression regions fell within the hot spot are identified in the legend.

**Fig 7:**
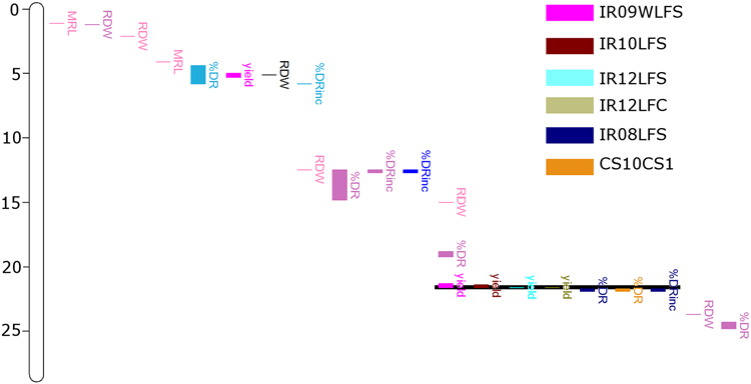
A genomic region on chromosome 8 (20.3–21.9 Mb) was identified as a hot spot in which percent deep roots and yield aligned. Colors indicate each experiment from which the phenotypic data and introgression regions were correlated. Experiments from which introgression regions fell within the hot spot are identified in the legend.

Chromosome maps of introgression blocks that were significantly correlated with root traits and grain yield under both drought stress and well-watered control treatment) revealed alignment of the greatest numbers of introgression blocks to two chromosome regions. One on chromosome 1 (39.7–40.7 Mb) in which root dry weight, percent deep roots, and grain yield aligned (Fig 6); and the other on chromosome 8 (20.3–21.9 Mb) in which percent deep roots and grain yield aligned (Fig 7). A database search and enrichment analysis identified a number of previously reported rice root QTLs for both of these chromosome regions (Table 6).

**Table 6:** Previously reported root-related QTLs within the regions of chromosomes 1 and 8 where the highest number of traits aligned in this study.

	Category	Trait	Gramene QTL ID	List hits	List total	Population hits	P value	Reference
Chr 1	Vigor	root number	DQC3	11	11	28	5.19E-25	[33]
	Vigor	root number	AQC003	11	11	28	5.19E-25	[33]
	Vigor	root number	AQO077	11	11	28	5.19E-25	[34]
	Vigor	root dry weight	AQGI070	11	11	42	1.03E-22	[35]
	Abiotic stress	root dry weight to tiller number ratio	CQQ13	9	11	56	1.21E-15	[36]
	Abiotic stress	root weight	CQQ6	9	11	56	1.21E-15	[36]
	Abiotic stress	root weight	CQQ32	9	11	56	1.21E-15	[37]
	Abiotic stress	penetrated root thickness	DQF9	9	11	56	1.21E-15	[38]
	Abiotic stress	relative root length	CQL2	2	11	2	8.12E-06	[39]
	Abiotic stress	relative root length	CQL1	2	11	2	8.12E-06	[39]
	Anatomy	seminal root length	CQS3	2	11	2	8.12E-06	[40]
	Abiotic stress	penetrated root length	AQGC035	3	11	60	0.00061969	[41]
	Abiotic stress	penetrated root thickness	AQGC022	3	11	60	0.00061969	[41]
Chr 8	Vigor	root to shoot ratio	AQO017	13	17	22	3.29E-28	[34, 42]
	Vigor	root to shoot ratio	AQO025	13	17	22	3.29E-28	[34, 42]
	Vigor	root number	CQAW26	17	17	97	3.35E-28	[43]
	Abiotic stress	relative root length	CQL9	14	17	42	3.76E-26	[39]
	Abiotic stress	relative root length	CQL8	14	17	42	3.76E-26	[39]
	Anatomy	root length	AQZ004	4	17	13	2.17E-07	[44]
	Abiotic stress	relative root length	AQZ008	4	17	13	2.17E-07	[44]
	Anatomy	root thickness	AQAL029	4	17	120	0.00184121	[45]

Gramene QTL ID: the Gramene database accession code; List hits: the number of 100kb blocks in the current introgression bin that intersect the Gramene QTL; List total: the total number of 100kb blocks in current QTL bin; Population hits: the total number of genome-wide 100kb introgression blocks for the current Gramene QTL category (out of a total of 3680 100kb introgression blocks for all Gramene QTLs; P value: probability based on a one-tailed Fisher's Exact Test; Reference: the publication in which the QTL was reported.

## Discussion

The results of this study emphasize the high degree of sensitivity of rice to environmental effects, and the identified genomic hot spots reflect the possibility of strong functional relationships between root growth and yield. This highlights the advantages of studying multiple whole-plant traits rather than individual components [46].

Of the three root traits analyzed, RDW appeared to be the most consistent according to: A) consistent trends in RDW response to drought stress in all study systems (Fig 5B), B) the lower percentage of variation due to environmental (E) effects (Table 5), and C) the more closely-grouped distribution of RDW in the biplot, as compared to the more dispersed distribution for MRL and %DR.

Across the range of field and container study systems in the current study, no consistent distinctions were observed among root traits. In a precursor to this study comparing the practicality of the protocols and genotypic performance in hydroponics, rhizotron, and non-woven fabric systems [19], it was concluded that the rhizotron system was most preferable, while it showed a distinct response for MRL in the present study in that it did not group with other experiments in the biplot (Fig 5C). Likewise, the greater increase in MRL by drought observed in lysimeter study IR08CC may have been due to the soil type and relatively smaller volume of the lysimeters compared to cylinder study Ba10C, in which a smaller increase in MRL by drought was observed (Fig 2C). The distinct response of the CS09CC and CS10CS cylinder studies for %DR (Fig 2D) may be related to the earlier growth stage at which drought stress was induced, as well as the tendency of that soil to become very resistant to root penetration upon drying. Both of these factors likely restricted root growth and resulted in the most severe drought stress being induced for this experiment (Fig 2A). Given the stronger correlations between trait PCA values and soil texture, it is apparent that the soil type/ growth medium had stronger relationships with the traits measured than the container dimensions.

Environmental conditions, methodologies, and the different characteristics of each experimental setup likely influenced the genotypic responses in each experiment relative to the other experiments. For example, the separate response of the TNAU root measurements in terms of absolute values (S1–S4 Tables) and clustering (Figs 4 and 5) may be due to a combination of methodology and site characteristics; while no distinct hard pan has been observed at that site, this was the only field site from which MRL was measured and thus may have resulted in different responses compared to the container studies in which MRL was measured. Studies conducted in the same environment tended to correlate (Table 4) and cluster with each other (Figs 4 and 5). The differences among experimental setups was most profound in the drought stress treatments, which showed fewer correlations with other experiments than the control treatments (Table 4), indicating that the level of drought stress achieved increased the magnitude of differences among experiments. The high number of experiments to which the Nagoya (Na10 and Na11) and the non-woven fabric (Ab09CNW) experiments were correlated may be due to the level of restriction of root growth that both systems applied which might have better discriminated among genotypes for RDW. Yet, in the case of Na10 and Na11 the lower number of genotypes evaluated may also have affected these observed correlations. Furthermore, the conditions to which each of the 20 diverse OryzaSNP genotypes are adapted (flooded vs aerobic) probably contributed to their varying responses across experiments.

Despite the OryzaSNP set being comprised of highly diverse genotypes, the genotype (G) component explained the smallest percent of variation for yield and root traits. OryzaSNP genotypes Dular, Azucena, and Moroberekan were previously classified as deep-rooted under drought in the field at IRRI and in lysimeters [20, 26]. In the hydroponics, rhizotrons, non-woven fabric, and herbicide placement at depth studies at the University of Aberdeen, cultivars Azucena and FR13A were notable for having long roots in hydroponics and rhizotrons while Nipponbare and IR64 were at the opposite extreme, and Dular was notable for having higher root length in hydroponics than in rhizotrons [19]. In addition to varying in absolute measurements, the OryzaSNP genotypes also varied in their response to drought stress (Fig 2) but these responses were not correlated among the three root traits studied, highlighting that different root parameters can be differentially sensitive to environmental characteristics. Regardless of phenology, genotypes with either constitutively deep roots or those that display deep roots only in response to drought may contribute to grain yield under drought, depending on the type of drought stress that occurs.

Since no general genotypic trends were apparent across the environments in this study, this highlights the importance of genetic variation within an environment, which was significant. The relative effects of G, E, and G x E are largely dependent on the chosen range of environments used, and drought stress can occur at a range of severities and growth stages within a given environment. The results from this study imply that rice responses to drought for yield and root growth would be best evaluated within the target environment and under the conditions similar to those in which they will be used. As such, the range of methods presented here could be used as a guide from which future rice root researchers could choose a method, based on the goals of the experiment, the target drought environment, and the resources available.

The lower E effect on yield compared to the root traits may reflect that A) all grain yield data were collected from field experiments (although in diverse environments) and root data were collected from a larger range of study systems, and B) root growth shows a higher sensitivity than grain yield to environmental conditions. A greater stability of grain yield to environmental variation than root traits may explain some of the previously reported lack of effects of root QTLs on grain yield [6, 47]. Furthermore, it is possible that additional root traits (hydraulics or root growth plasticity, for example) may also be involved in the contribution of roots to grain yield. More work is needed to know what root traits contribute most to grain yield in different environments before those traits can be targeted for selection in drought breeding programs.

Despite the strong E effect, the two genomic hot spots where multiple traits aligned on Chromosomes 1 and 8 were observed from a range of study systems; these traits included grain yield from field studies, root growth from field studies, and root traits from container systems (Figs 6 and 7). The ability to identify significant introgression regions for these traits in such a relatively small set of genotypes may be due to the fact that the 20 OryzaSNP genotypes are so diverse, and because they were characterized in such a range of conditions in this study. The co-location of root traits from a multiple study systems, as well as the correlations between study systems (Table 4) further indicates that container root study systems do represent responses that can be relevant to yield under drought stress from field studies. Such agreement of root trait-associated genomic regions may in fact be better indicators of the effectiveness of a particular screening method that comparison with grain yield, although root traits measured from greenhouse experiments have previously shown positive correlations with yield under drought in rice [22, 48–51].

The hot spot on chromosome 1 identified in this study falls within one of the six hot spots identified from a meta-analysis of 1,467 QTLs reported to be related to root traits in rice [11]. This is a region identified in the meta-analysis of the Bala x Azucena mapping population [52] as having activity for root traits and leaf morphology traits, and it is below the meta-QTL for drought avoidance and plant height associated with the semi-dwarf gene *sd1* located at 38.4 Mbp. The region on chromosome 1 was also similar to a locus significantly associated with root angle in a tropical japonica panel grown in a glass bead-based “Rhizoscope” system [53], and was a close match to one of four key regions for drought response traits [10]. In addition to the root QTLs common to this region, the region on chromosome 1 in this study is near the major-effect drought-yield QTL *qDTY*_*1*.*1*_ [54] and other QTL for several drought response traits [23]. Taken together, these observations and our results highlight the importance of this region for drought response and suggest that root growth at depth should be evaluated as part of the physiological dissection of these QTL.

The hot spot on Chromosome 8 identified in this study co-locates with a key region for drought response in rice, in which more than 30 QTLs for traits including grain yield, plant type, spikelet fertility, and drought response traits including osmotic adjustment, cell membrane stability, and leaf relative water content have been reported [10]. In the Bala x Azucena population, the region of Chromosome 8 around 20–21 Mbp was identified as a meta-QTL for drought avoidance [52] and a QTL for grain yield under stress in Coimbatore, India [55].

Unlike the recently published rice root GWAS study [53], the current study was not an association analysis of independent SNP to phenotypes. Rather, our analysis identified correlations between traits and blocks indicative of introgression from one type into background genotypes. That is, the analysis used the 100-kb blocks from one type (subgroup) that are introgressed into the background of a different subgroup (e.g. japonica-type regions in the background of aus or indica genotypes) identified by [16]. This constitutes a meta-analysis whereby hypotheses can be formulated, e.g. do these regions contain candidate genes and potential modules for yield under drought that may be co-regulated via epigenomic and chromatin architecture?

The higher frequency of japonica introgression regions that were correlated with yield and root traits in this study may be due to 1) the effect of having an introgression block data set where introgressions were identified by SNP relative to only the japonica reference genome variety (Nipponbare), that reduced the number and location of putative introgression blocks compared to that of the aus or indica types, and 2) the deeper and coarser types of roots of japonica genotypes [5]—the root type that was screened for in most experiments in this study. These results are preliminary and subject to further experiments and validation, but they suggest that the genomes of some of the deep-rooted aus or indica genotypes in the OryzaSNP panel may have outcrossed with japonica genotypes at some point in time, contributing to the deep-rooted phenotype expressed in some japonica genotypes—particularly the tropical and upland japonica.

## Conclusions

A better understanding of the physiological and genetic components behind rice performance under drought will help guide approaches to improving rice response to drought. In an evaluation of rice drought response for yield and root growth where data were obtained from 37 environments and from 20 diverse genotypes, the GxE effect was lower for root traits than for grain yield, and no single genotype was significant in terms of performance across all environments. This confirms the high degree of sensitivity of rice to its environmental conditions and suggests that genotypic drought screening will be most effective when conducted in the targeted environmental conditions with germplasm that has some adaptation to these environments. However, the identification of two genomic hot spots at which 20 environments/traits aligned points to the possibility for improvement of the stability of rice drought response to diverse environmental conditions. The co-location of root traits from a range of study systems indicates the potential utility of container root study systems to represent responses that can be relevant to yield in field studies. Since the identified genomic regions co-located with QTL and meta-QTL for a number of previously reported root traits and also for a major-effect QTL for yield under drought, these regions are good candidates for detailed characterization to contribute to understanding the improvement of rice response to drought.

## Supporting Information

S1 Table Least squares mean values for grain yield (kg ha^-1^) across sites. Data previously reported by Henry et al (2011) are included in this table.
(DOCX)

S2 Table Least squares mean values for root dry weight (g) across sites. Data previously reported by Henry et al (2011), Gowda et al (2012), and Shrestha et al (2013) are included in this table.
(DOCX)

S3 Table Least squares mean values for maximum root depth (cm) across sites. Data previously reported by Gowda et al (2012) and Shrestha et al (2013) are presented in this table.
(DOCX)

S4 Table Least squares mean values for % deep roots across sites. Data previously reported by Henry et al (2011), Gowda et al (2012), and Shrestha et al (2013) are included in this table.
(DOCX)

S5 Table Correlation matrix for grain yield among experiments. * = p<0.05, ** = p<0.01, *** = p<0.001. Data previously reported by Henry et al (2011) were used to calculate some of the results shown in this table.
(DOCX)

S6 Table Correlation matrix for root dry weight among experiments. * = p<0.05, ** = p<0.01, *** = p<0.001. Data previously reported by Henry et al (2011), Gowda et al (2012), and Shrestha et al (2013) were used to calculate some of the results shown in this table.
(DOCX)

S7 Table Correlation matrix for maximum root depth among experiments. * = p<0.05, ** = p<0.01, *** = p<0.001. Data previously reported by Gowda et al (2012), and Shrestha et al (2013) were used to calculate some of the results shown in this table.
(DOCX)

S8 Table Correlation matrix for percent deep roots among experiments. * = p<0.05, ** = p<0.01, *** = p<0.001. Data previously reported by Henry et al (2011), Gowda et al (2012), and Shrestha et al (2013) were used to calculate some of the results shown in this table.
(DOCX)

S9 Table Results from AMMI analysis using only experiments in which both yield and root traits (RDW and MRL) were measured. The analysis could not be conducted for %DR because only two experiments measured both yield and %DR.
(DOCX)

S10 Table Correlations among the first two principle components for each trait with the environmental characteristics of the experiments. Significant correlations are indicated by *<0.05, **<0.01, and ***<0.001.
(DOCX)

S11 Table Introgression regions detected to correlate with traits phenotyped in this study in the OryzaSNP panel. (DOCX)

S1 Fig Analysis including the subset of 7 OryzaSNP genotypes phenotyped for RDW in the line-source system at Nagoya Univ. (DOCX)

## References

[1] Pandey S, Bhandari H. Drought: economic costs and research implications In: Serraj R., Bennett J., Hardy B., editors. Drought Frontiers in Rice: Crop Improvement for Increased Rainfed Production. Singapore: World Scientific Publishing and Los Baños (Philippines): International Rice Research Institute; 2008 10.3389/fphys.2012.00429

[2] Serraj R, McNally KL, Slamet-Loedin I, Kohli A, Haefele SM, Atlin G, et al Drought resistance improvement in rice: an integrated genetic and resource management strategy. Plant Prod Sci. 2011;14: 1–14.

[3] Serraj R, Kumar A, McNally KL, Slamet-Loedin I, Bruskiewich R, Mauleon R, et al Improvement of drought resistance in rice. Adv Agron. 2009;103: 41–98.

[4] Chandra Babu R, Shashidhar HE, Lilley JM, Thanh ND, Ray JD, Sadasivam S, et al Genetic variation in root penetration ability, osmotic adjustment and dehydration tolerance among rice lines adapted to rainfed lowland and upland ecosystems. Plant Breeding 2001;120: 233–238.

[5] Lafitte HR, Champoux MC, McLaren G, O’Toole JC. Rice root morphological traits are related to isozyme group and adaptation. Field Crop Res. 2001;71: 57–70.

[6] Venuprasad R, Shashidhar HE, Hittalmani S, Hemamalini GS. Tagging quantitative trait loci associated with grain yield and root morphological traits in rice (*Oryza sativa* L.) under contrasting moisture regimes. Euphytica. 2002;128: 293–300.

[7] Srinivasan S, Gomez SM, Kumar SS, Ganesh SK, Biji KR, Senthil A, et al QTLs linked to leaf epicuticular wax, physio-morphological and plant production traits under drought stress in rice (*Oryza sativa* L.). Plant Growth Regul. 2008;56: 245–256.

[8] Nguyen HT, Chandra Babu R, Blum A. Breeding for drought resistance in rice: Physiology and molecular genetics considerations. Crop Sci. 1997;37: 1426–1434.

[9] Samson BK, Hasan M, Wade LJ. Penetration of hardpans by rice lines in the rainfed lowlands. Field Crop Res. 2002;76: 175–188.

[10] Kamoshita A, Babu RC, Boopathi NM, Fukai S. Phenotypic and genotypic analysis of drought-resistance traits for development of rice cultivars adapted to rainfed environments. Field Crop Res. 2008;109: 1–23.

[11] Courtois B, Ahmadi N, Khowaja F, Price AH, Rami J-F, et al Rice root genetic architecture: meta-analysis from a drought QTL database. Rice. 2009; 2: 115–128. 10.1007/s12284-009-9028-9

[12] Gowda VRP, Henry A, Yamauchi A, Shashidhar HE, Serraj R. Root biology and genetic improvement for drought avoidance in rice. Field Crop Res. 2011;122: 1–13.

[13] Wade LJ, McLaren CG, Quintana L, Harnpichitvitaya D, Rajatasereekul S, Sarawgi AK, et al Genotype by environment interactions across diverse rainfed lowland rice environments. Field Crop Res. 1999;64: 35–50.

[14] Cooper M, Rajatasereekul S, Immark S, Fukai S, Basnayake J. Rainfed lowland rice breeding strategies for northeast Thailand. I. Genotypic variation and genotype x environment interactions for grain yield. Field Crop Res. 1999;64: 131–151.

[15] Acuña TLB, Lafitte HR, Wade LJ. Genotype x environment interactions for grain yield of upland rice backcross lines in diverse hydrological environments. Field Crop Res. 2008;108: 117–125.

[16] McNally KL, Childs KL, Bohnert R, Davidson RM, Zhao K, Ulat VJ, et al Genomewide SNP variation reveals relationships among landraces and modern varieties of rice. P Natl Acad Sci USA. 2009;106: 12273–12278. 10.1073/pnas.0900992106 19597147 PMC2718348

[17] Jahn CE, Mckay JK, Mauleon R, Stephens J, McNally KL, Bush DR, et al Genetic variation in biomass traits among 20 diverse rice varieties. Plant Physiol. 2010;155: 157–168. 10.1104/pp.110.165654 21062890 PMC3075782

[18] Shashidhar HE, Henry A, Hardy B, eds: Methodologies for root drought studies in rice. International Rice Research Institute, Los Baños, Philippines; 2012.

[19] Shrestha R, Al-Shugeairy Z, Al-Ogaidi F, Munasinghe M, Radermacher M, Vandenhirtz J, et al Comparing simple root phenotyping methods on a core set of rice genotypes. Plant Biology. 2013;16: 632–642. 10.1111/plb.12096 24015692

[20] Gowda VRP, Henry A, Vadez V, Shashidhar HE, Serraj R. Water uptake dynamics under progressive drought stress drought stress in OryzaSNP panel rice accessions. Funct Plant Biol. 2012;39: 402–411. 10.1071/FP12015 32480792

[21] Pasuquin EM, Hasegawa T, Eberbach P, Reinke R, Wade LJ, Lafarge T. Responses of eighteen rice (*Oryza sativa* L.) cultivars to temperature tested two types of growth chambers. Plant Prod Sci. 2013;16: 217–225.

[22] Chandra Babu R, Nguyen BD, Chamarerk V, Shanmugasundaram P, Chezhian P, Jeyaprakash P, et al Genetic analysis of drought resistance in rice by molecular markers: Association between secondary traits and field performance. Crop Sci. 2003;43: 1457–1469. 14502478

[23] Arvindkumar SS, Poornima SR, Silvas K, Prince J, Kanagaraj P, Sheeba JA, et al Fine mapping QTL for drought resistance traits in rice (*Oryza sativa* L.) using bulk segregant analysis. Mol Biotechnol. 2011;49: 90–95. 10.1007/s12033-011-9382-x 21298364

[24] Gomez SM, Boopathi NM, Kumar SS, Ramasubramanian T, Chengsong Z, Jeyaprakash P, et al Molecular mapping and location of QTLs for drought-resistance traits in indica rice (*Oryza sativa* L.) lines adapted to target environments. Acta Physiol Plant. 2010;32: 355–364.

[25] Suji KK, Silvas K, Prince J, Mankhar PS, Kanagaraj P, Poornima R, et al Evaluation of rice (*Oryza sativa* L.) near iso-genic lines with root QTLs for plant production and root traits in rainfed target populations of environment. Field Crop Res. 2012;137: 89–96.

[26] Henry A, Gowda VRP, Torres R, McNally K, Serraj R. Variation in root system architecture and drought response in rice (*Oryza sativa*): Phenotyping of the OryzaSNP panel in rainfed lowland fields. Field Crop Res. 2011;120: 205–214.

[27] Kano NM, Inukai Y, Wade LJ, Siopongco JDLC, Yamauchi A. Root development, water uptake, and shoot dry matter production under water deficit conditions in two CSSLs of rice: functional roles of root plasticity. Plant Prod Sci. 2011;14: 307–317.

[28] SAS Institute Inc. SAS OnlineDoc, Version 9.3. SAS Institute Inc, Cary, NC; 2012.

[29] R Core Team. R: A language and environment for statistical computing. R Foundation for Statistical Computing Vienna, Austria; 2013 10.3758/s13428-013-0330-5

[30] IRRI. CropStat for Windows, version 7.2. Available at http://bbi.irri.org. Biometrics and Breeding Informatics, PBGB, International Rice Research Inst., Los Baños, Laguna, Philippines; 2007.

[31] Ouyang S, Zhu W, Hamilton J, Lin H, Campbell M, Childs K, et al The TIGR rice genome annotation resource: improvements and new features. Nucleic Acids Res. 2007;35(Database issue): D883–D887. 17145706 10.1093/nar/gkl976 PMC1751532

[32] Voorrips RE. MapChart Software for the graphical presentation of linkage maps and QTLs. J Hered. 2002; 93: 77–78. 12011185 10.1093/jhered/93.1.77

[33] Price AH, Steele KA, Moore BJ, Barraclough PB, Clark LJ. A combined RFLP and AFLP linkage map of upland rice (*Oryza sativa* L.) used to identify QTLs for root-penetration ability. Theor Appl Genet. 2000;100: 49–56.

[34] Price AH, Steele KA, Moore BJ, Jones RGW. Upland rice grown in soil-filled chambers and exposed to contrasting water-deficit regimes II. Mapping quantitative trait loci for root morphology and distribution. Field Crop Res. 2002;76: 25–43.

[35] Lian X, Xing Y, Yan H, Xu C, Li X, Zhang Q. QTLs for low nitrogen tolerance at seedling stage identified using a recombinant inbred line population derived from an elite rice hybrid. Theor Appl Genet. 2005;112: 85–96. 16189659 10.1007/s00122-005-0108-y

[36] Yadav R, Courtois B, Huang N, McLaren G. Mapping genes controlling root morphology and root distribution in a doubled-haploid population of rice. Theor Appl Genet. 1997;94: 619–632.

[37] Shen L, Courtois B, McNally KL, Robin S, Li Z. Evaluation of near-isogenic lines of rice introgressed with QTLs for root depth through marker-aided selection. Theor Appl Genet. 2001;103: 75–83.

[38] Zheng HG, Babu RC, Pathan MS, Ali L, Huang N, Courtois B, et al Quantitative trait loci for root-penetration ability and root thickness in rice: comparison of genetic backgrounds. Genome (National Research Council Canada). 2000;43: 53–61. 10701113

[39] Wu P, Liao CY, Hu B, Yi KK, Jin WZ, Ni JJ, et al QTLs and epistasis for aluminum tolerance in rice (*Oryza sativa* L.) at different seedling stages. Theor Appl Genet. 2000;100: 1295–1303.

[40] Zhang WP, Shen XY, Wu P, Hu B, Liao CY QTLs and epistasis for seminal root length under a different water supply in rice (*Oryza sativa* L.). Theor Appl Genet. 2001;103: 118–123.

[41] Ali ML, Pathan MS, Zhang J, Bai G, Sarkarung S, Nguyen HT. Mapping QTLs for root traits in a recombinant inbred population from two indica ecotypes in rice. Theor Appl Genet. 2000;101: 756–766.

[42] Price AH, Steele KA, Gorham J, Bridges JM, Moore BJ, Evans JL, et al Upland rice grown in soil-filled chambers and exposed to contrasting water-deficit regimes I. Root distribution, water use and plant water status. Field Crop Res. 2002;76: 11–24.

[43] Ray JD, Yu L, McCouch SR, Champoux MC, Wang GL, Nguyen HT. Mapping quantitative trait loci associated with root penetration ability in rice. Theor Appl Genet. 1996;92: 627–636. 10.1007/BF00226082 24166384

[44] Nguyen BD, Brar DS, Bui BC, Nguyen TV, Pham LN, Nguyen HT. Identification and mapping of the QTL for aluminum tolerance introgressed from the new source, ORYZA RUFIPOGON Griff., into indica rice (*Oryza sativa* L.). Theor Appl Genet. 2003;106: 583–593. 12595985 10.1007/s00122-002-1072-4

[45] Kamoshita A, Wade LJ, Ali L, Pathan S, Zhang J, Sarkarung S, et al Mapping QTLs for root morphology of a rice population adapted to rainfed lowland conditions. Theor Appl Genet. 2002;104: 880–893. 12582650 10.1007/s00122-001-0837-5

[46] Shashidhar HE, Kanbar A, Toorchi M, Raveendra GM, Kundur P, Vimarsha HS, et al Breeding for drought resistance based on whole plant architecture: Conventional and molecular approaches. Plant Breeding from Laboratories to Field, Intech Publishers; 2013 pp.151–166. 10.5772/54983

[47] Price AH, Cairns JE, Horton P, Jones HG and Griffiths H. Linking drought resistance mechanisms to drought avoidance in upland rice using a QTL approach; progress and new opportunities to integrate stomatal and mesophyll responses. J Exp Bot. 2002;53: 989–1004. 11971911 10.1093/jexbot/53.371.989

[48] Wade LJ, Kamoshita A, Yamauchi A, Azhiri-Sigari T. Genotypic variation in response of rainfed lowland rice to drought and rewatering, 1. Growth and water use. Plant Prod Sci. 2000;3: 173–179.

[49] Siopongco J, Yamauchi A, Salekdeh H, Bennet J, Wade LJ. Root growth and water extraction response of doubled-haploid rice lines to drought and rewatering during the vegetative stage. Plant Prod Sci. 2005;8: 497–508.

[50] Cairns JE, Audebert A, Mullins CE, Price AH. Mapping quantitative trait loci associated with root growth in upland rice (*Oryza sativa* L.) exposed to soil water-deficit in fields with contrasting soil properties. Field Crop Res. 2009;114: 108–118.

[51] Kano-Nakata M, Gowda VRP, Henry A, Serraj R, Inukai Y, Fujita D, et al Functional roles of the plasticity of root system development in biomass production and water uptake under rainfed lowland conditions. Field Crop Res. 2013;144: 288–296.

[52] Khowaja FS, Norton GJ, Courtois B, Price AH. Improved resolution in the position of drought-related QTLs in a single mapping population of rice by meta-analysis. BMC Genomics. 2009;10: 276 10.1186/1471-2164-10-276 19545420 PMC2708188

[53] Courtois B, Audebert A, Dardou A, Roques S, Ghneim HT, Droc G, et al Genome-wide association mapping of root traits in a japonica rice panel. PLoS ONE. 2013;8: e78037 10.1371/journal.pone.0078037 24223758 PMC3818351

[54] Vikram P, Mallikarjuna Swamy BP, Dixit S, Ahmed HU, Sta Cruz MT, Singh AK, et al DTY1.1, a major QTL for rice grain yield under reproductive-stage drought stress with a consistent effect in multiple elite genetic backgrounds. BMC Genetics. 2011;12: 89 10.1186/1471-2156-12-89 22008150 PMC3234187

[55] Gomez MS, Satheesh Kumar S, Jeyaprakash P, Suresh R, Biji KR, Manikanda Boopathi N, et al Mapping QTLs linked to physio-morphological and plant production traits under drought stress in rice (*Oryza sativa* L.) in the target environment. Amer J Biochem Biotechnol. 2006;2: 161–169.

