# Correction: Environmental Response and Genomic Regions Correlated with Rice Root Growth and Yield under Drought in the OryzaSNP Panel across Multiple Study Systems

**DOI:** 10.1371/journal.pone.0335770

**Published:** 2025-10-29

**Authors:** Len J. Wade, Violeta Bartolome, Ramil Mauleon, Vivek Deshmuck Vasant, Sumeet Prabhakar Mankar, Muthukumar Chelliah, Emi Kameoka, K. Nagendra, K. R. Kamalnath Reddy, C. Mohan Kumar Varma, Kalmeshwar Gouda Patil, Roshi Shrestha, Zaniab Al-Shugeairy, Faez Al-Ogaidi, Mayuri Munasinghe, Veeresh Gowda, Mande Semon, Roel R. Suralta, Vinay Shenoy, Vincent Vadez, Rachid Serraj, H. E. Shashidhar, Akira Yamauchi, Ranganathan Chandra Babu, Adam Price, Kenneth L. McNally, Amelia Henry

The fifth author’s name is spelled incorrectly. The correct name is: Sumeet Prabhakar Mankar. The correct citation is: Wade LJ, Bartolome V, Mauleon R, Vasant VD, Mankar SP, Chelliah M, et al. (2015) Environmental Response and Genomic Regions Correlated with Rice Root Growth and Yield under Drought in the OryzaSNP Panel across Multiple Study Systems. PLoS ONE 10(4): e0124127. https://doi.org/10.1371/journal.pone.0124127.

## References

[1] Wade LJ, Bartolome V, Mauleon R, Vasant VD, Prabakar SM, Chelliah M, et al. Environmental Response and Genomic Regions Correlated with Rice Root Growth and Yield under Drought in the OryzaSNP Panel across Multiple Study Systems. PLoS One. 2015;10(4):e0124127. doi: 10.1371/journal.pone.0124127 25909711 PMC4409324

