# Supplementary material for: Environmental Response and Genomic Regions Correlated with Rice Root Growth and Yield under Drought in the OryzaSNP Panel across Multiple Study Systems
Source: PLoS One. 2015 Apr 24;10(4):e0124127. doi: 10.1371/journal.pone.0124127 (PMC4409324; doi:10.1371/journal.pone.0124127)
Supplement: S1 Table — Data previously reported by Henry et al (2011) are included in this table. (DOCX) [file pone.0124127.s001.docx]

**S1 Table. Least squares mean values for grain yield (kg ha^-1^) across sites.** Data previously reported by Henry et al (2011) are included in this table.

^a^ based on single-plant measurements in two replicates
