# Supplementary material for: Environmental Response and Genomic Regions Correlated with Rice Root Growth and Yield under Drought in the OryzaSNP Panel across Multiple Study Systems
Source: PLoS One. 2015 Apr 24;10(4):e0124127. doi: 10.1371/journal.pone.0124127 (PMC4409324; doi:10.1371/journal.pone.0124127)
Supplement: S3 Table — Data previously reported by Gowda et al (2012) and Shrestha et al (2013) are presented in this table. (DOCX) [file pone.0124127.s003.docx]

**S3 Table. Least squares mean values for maximum root depth (cm) across sites.** Data previously reported by Gowda et al (2012) and Shrestha et al (2013) are presented in this table.
