# Supplementary material for: Environmental Response and Genomic Regions Correlated with Rice Root Growth and Yield under Drought in the OryzaSNP Panel across Multiple Study Systems
Source: PLoS One. 2015 Apr 24;10(4):e0124127. doi: 10.1371/journal.pone.0124127 (PMC4409324; doi:10.1371/journal.pone.0124127)
Supplement: S4 Table — Data previously reported by Henry et al (2011), Gowda et al (2012), and Shrestha et al (2013) are included in this table. (DOCX) [file pone.0124127.s004.docx]

**S4 Table. Least squares mean values for % deep roots across sites.** Data previously reported by Henry et al (2011), Gowda et al (2012), and Shrestha et al (2013) are included in this table.
