# Supplementary material for: Environmental Response and Genomic Regions Correlated with Rice Root Growth and Yield under Drought in the OryzaSNP Panel across Multiple Study Systems
Source: PLoS One. 2015 Apr 24;10(4):e0124127. doi: 10.1371/journal.pone.0124127 (PMC4409324; doi:10.1371/journal.pone.0124127)
Supplement: S5 Table — * = p<0.05, ** = p<0.01, *** = p<0.001. Data previously reported by Henry et al (2011) were used to calculate some of the results shown in this table. (DOCX) [file pone.0124127.s005.docx]

**S5 Table. Correlation matrix for grain yield among experiments.** * = p<0.05, **=p<0.01, *** = p<0.001. Data previously reported by Henry et al (2011) and Gowda et al (2012) were used to calculate some of the results shown in this table. Data previously reported by Henry et al (2011) were used to calculate some of the results shown in this table.
