# Supplementary material for: Environmental Response and Genomic Regions Correlated with Rice Root Growth and Yield under Drought in the OryzaSNP Panel across Multiple Study Systems
Source: PLoS One. 2015 Apr 24;10(4):e0124127. doi: 10.1371/journal.pone.0124127 (PMC4409324; doi:10.1371/journal.pone.0124127)
Supplement: S6 Table — * = p<0.05, ** = p<0.01, *** = p<0.001. Data previously reported by Henry et al (2011), Gowda et al (2012), and Shrestha et al (2013) were used to calculate some of the results shown in this table. (DOCX) [file pone.0124127.s006.docx]

**S6 Table. Correlation matrix for root dry weight among experiments.** * = p<0.05, **=p<0.01, *** = p<0.001. Data previously reported by Henry et al (2011), Gowda et al (2012), and Shrestha et al (2013) were used to calculate some of the results shown in this table.
