# Supplementary material for: Environmental Response and Genomic Regions Correlated with Rice Root Growth and Yield under Drought in the OryzaSNP Panel across Multiple Study Systems
Source: PLoS One. 2015 Apr 24;10(4):e0124127. doi: 10.1371/journal.pone.0124127 (PMC4409324; doi:10.1371/journal.pone.0124127)
Supplement: S7 Table — * = p<0.05, ** = p<0.01, *** = p<0.001. Data previously reported by Gowda et al (2012), and Shrestha et al (2013) were used to calculate some of the results shown in this table. (DOCX) [file pone.0124127.s007.docx]

**S7 Table. Correlation matrix for maximum root depth among experiments.** * = p<0.05, **=p<0.01, *** = p<0.001. Data previously reported by Gowda et al (2012), and Shrestha et al (2013) were used to calculate some of the results shown in this table.

|  | **Ab09CH** | **Ab09CR** | **Ba10CC** | **Ba10CS** | **IR08CC** | **IR08CS** | **TN10FC** | **TN10FS** | **TN11FC** |
| --- | --- | --- | --- | --- | --- | --- | --- | --- | --- |
| **Ab09CR** | 0.47 |  |  |  |  |  |  |  |  |
| **Ba10CC** | 0.48 | 0.54* |  |  |  |  |  |  |  |
| **Ba10CS** | 0.48 | 0.35 | 0.85*** |  |  |  |  |  |  |
| **IR08CC** | 0.19 | 0.51* | 0.5* | 0.38 |  |  |  |  |  |
| **IR08CS** | 0.35 | 0.41 | 0.61** | 0.49* | 0.4 |  |  |  |  |
| **TN10FC** | 0.62 | 0.59* | 0.37 | 0.33 | 0.17 | 0.35 |  |  |  |
| **TN10FS** | 0.71* | 0.41 | 0.38 | 0.05 | 0.42 | 0.42 | 0.36 |  |  |
| **TN11FC** | 0.28 | 0.09 | 0.4 | 0.28 | 0.43 | 0.47 | 0.14 | 0.5 |  |
| **TN11FS** | 0.69* | 0.25 | 0.4 | 0.22 | 0.18 | 0.02 | 0.25 | 0.42 | 0.37 |
