# Supplementary material for: Environmental Response and Genomic Regions Correlated with Rice Root Growth and Yield under Drought in the OryzaSNP Panel across Multiple Study Systems
Source: PLoS One. 2015 Apr 24;10(4):e0124127. doi: 10.1371/journal.pone.0124127 (PMC4409324; doi:10.1371/journal.pone.0124127)
Supplement: S8 Table — * = p<0.05, ** = p<0.01, *** = p<0.001. Data previously reported by Henry et al (2011), Gowda et al (2012), and Shrestha et al (2013) were used to calculate some of the results shown in this table. (DOCX) [file pone.0124127.s008.docx]

**S8 Table. Correlation matrix for percent deep roots among experiments.** * = p<0.05, **=p<0.01, *** = p<0.001. Data previously reported by Henry et al (2011), Gowda et al (2012), and Shrestha et al (2013) were used to calculate some of the results shown in this table.

|  | **Ab09CH** | **Ab09CNW** | **Ab09CR** | **CS09CC** | **CS10CS1** | **CS10CS2** | **IR08CC** | **IR08CS** | **IR08FLC** |
| --- | --- | --- | --- | --- | --- | --- | --- | --- | --- |
| **Ab09CNW** | -0.47 |  |  |  |  |  |  |  |  |
| **Ab09CR** | -0.48 | 0.53* |  |  |  |  |  |  |  |
| **CS09CC** | 0.46 | -0.44 | -0.48* |  |  |  |  |  |  |
| **CS10CS1** | 0.42 | -0.02 | -0.03 | 0.25 |  |  |  |  |  |
| **CS10CS2** | 0.52 | -0.58* | -0.43 | 0.27 | 0.1 |  |  |  |  |
| **IR08CC** | -0.53* | 0.41 | 0.55* | -0.4 | 0.04 | -0.23 |  |  |  |
| **IR08CS** | -0.23 | 0.14 | 0.3 | 0.03 | -0.17 | -0.04 | 0.51* |  |  |
| **IR08LFC** | 0.33 | 0.13 | -0.11 | 0.2 | 0.61* | 0.02 | 0.16 | -0.02 |  |
| **IR08LFS** | -0.29 | 0.33 | 0.38 | -0.4 | -0.44 | 0.11 | 0.52* | 0.43 | -0.15 |
