# Supplementary material for: Environmental Response and Genomic Regions Correlated with Rice Root Growth and Yield under Drought in the OryzaSNP Panel across Multiple Study Systems
Source: PLoS One. 2015 Apr 24;10(4):e0124127. doi: 10.1371/journal.pone.0124127 (PMC4409324; doi:10.1371/journal.pone.0124127)
Supplement: S9 Table — The analysis could not be conducted for %DR because only two experiments measured both yield and %DR. (DOCX) [file pone.0124127.s009.docx]

**S9 Table. Results from AMMI analysis using only experiments in which both yield and root traits (RDW and MRL) were measured.** The analysis could not be conducted for %DR because only two experiments measured both yield and %DR.

|  | Grain yield | RDW |  | Grain yield | MRL |
| --- | --- | --- | --- | --- | --- |
| # of Experiments: | 7 | |  | 4 | |
| Source of variation | % variation from Total SS | | | | |
| G | 14.6 | 18.0 |  | 19.7 | 45.5 |
| E | 35.1 | 53.1 |  | 44.3 | 7.1 |
| GxE | 50.3 | 28.9 |  | 36.0 | 47.4 |
