# Supplementary material for: Environmental Response and Genomic Regions Correlated with Rice Root Growth and Yield under Drought in the OryzaSNP Panel across Multiple Study Systems
Source: PLoS One. 2015 Apr 24;10(4):e0124127. doi: 10.1371/journal.pone.0124127 (PMC4409324; doi:10.1371/journal.pone.0124127)
Supplement: S10 Table — Significant correlations are indicated by *<0.05, **<0.01, and ***<0.001. (DOCX) [file pone.0124127.s010.docx]

**S10 Table. Correlations among the first two principle components for each trait with the environmental characteristics of the experiments.** Significant correlations are indicated by *<0.05, **<0.01, and ***<0.001.

|  | Soil volume | %Sand | %Silt | %Clay | Soil pH | Bulk density | Rainfall | Evaporation | Temp | Solar radiation | Relative humidity |
| --- | --- | --- | --- | --- | --- | --- | --- | --- | --- | --- | --- |
| yield PCA1 | - | -0.53 | 0.42 | 0.05 | 0.08 | -0.66 | 0.07 | -0.03 | -0.14 | -0.03 | 0.18 |
| yield PCA2 | - | -0.46 | 0.24 | 0.21 | 0.31 | 0.08 | 0.09 | -0.09 | -0.01 | -0.12 | 0.13 |
| RDW PCA1 | -0.8** | 0.7* | -0.33 | -0.4 | -0.25 | -0.06 | -0.25 | -0.92* | 0.02 | -0.34 | 0.4 |
| RDW PCA2 | -0.48 | -0.54 | 0.08 | 0.47 | -0.68** | 0.56 | 0.17 | -0.58 | -0.29 | 0.2 | -0.33 |
| MRL PCA1 | -0.8 | 0.9* | -0.9* | -0.9* | -0.69 | -0.99 | 0.04 | - | -0.05 | -0.8* | 0.73 |
| MRL PCA2 | -0.61 | 0.99** | -0.99** | -0.99** | 0.35 | 0.89 | -0.27 | - | 0.6 | -0.72 | 0.98*** |
| %DR PCA1 | 0.03 | -0.96* | 0.96* | -0.96* | -0.45 | -0.68 | - | - | 0.74* | 0.52 | -0.6 |
| %DR PCA2 | 0.08 | 0.53 | -0.53 | 0.53 | -0.09 | 0.18 | - | - | -0.07 | -0.04 | 0.15 |
