# Supplementary material for: Environmental Response and Genomic Regions Correlated with Rice Root Growth and Yield under Drought in the OryzaSNP Panel across Multiple Study Systems
Source: PLoS One. 2015 Apr 24;10(4):e0124127. doi: 10.1371/journal.pone.0124127 (PMC4409324; doi:10.1371/journal.pone.0124127)
Supplement: S11 Table — (DOCX) [file pone.0124127.s011.docx]

**S11 Table**. Introgression regions detected to correlate with traits phenotyped in this study in the OryzaSNP panel.

| Trait | Expt | Chrom | Subgroup | Min of s_block | Max of s_block |
| --- | --- | --- | --- | --- | --- |
| %DR | IR08FLC | 1 | aus | 11.1 | 11.2 |
|  |  | 7 | aus | 29.3 | 29.6 |
|  | IR08FLS | 8 | japonica | 21.7 | 21.9 |
|  | Ab09CNW | 3 | japonica | 15.6 | 15.6 |
|  |  | 7 | japonica | 22.5 | 22.7 |
|  |  | 10 | japonica | 11.7 | 11.7 |
|  | IR08CS | 1 | japonica | 19.7 | 19.8 |
|  |  | 2 | japonica | 3.5 | 3.8 |
|  |  | 4 | japonica | 33.9 | 34.4 |
|  |  | 5 | japonica | 17.5 | 17.9 |
|  |  | 8 | japonica | 12.5 | 14.8 |
|  |  |  | japonica | 18.8 | 19.2 |
|  |  |  | japonica | 24.3 | 24.8 |
|  |  | 10 | japonica | 10.9 | 10.9 |
|  | CS10CS1 | 8 | japonica | 21.7 | 21.9 |
|  | CS10CS2 | 1 | japonica | 20.2 | 20.4 |
|  |  |  | japonica | 22.5 | 23.1 |
|  |  |  | japonica | 36.8 | 37.4 |
|  |  |  | japonica | 38.6 | 39 |
|  |  |  | japonica | 40.2 | 40.2 |
|  |  |  | japonica | 41.4 | 41.7 |
|  |  | 3 | japonica | 7.2 | 7.5 |
|  |  |  | japonica | 9 | 9.8 |
|  |  | 4 | japonica | 6.5 | 7.4 |
|  |  |  | japonica | 22 | 22 |
|  |  |  | japonica | 25.1 | 25.1 |
|  |  |  | japonica | 27.1 | 27.2 |
|  |  |  | japonica | 28.4 | 29.8 |
|  |  | 5 | japonica | 5.8 | 5.9 |
|  |  |  | japonica | 29.2 | 29.7 |
|  |  | 6 | japonica | 5.6 | 5.6 |
|  |  |  | japonica | 6.6 | 7.3 |
|  |  | 7 | japonica | 25.8 | 26.4 |
|  |  | 8 | japonica | 4.4 | 5.8 |
|  |  | 10 | japonica | 0.2 | 3.5 |
|  |  | 12 | japonica | 23 | 24.8 |
|  |  |  | japonica | 25.8 | 26 |
| %DR inc | IR08FL | 8 | japonica | 21.7 | 21.9 |
|  | IR08C | 8 | japonica | 12.5 | 12.7 |
|  | IC09C | 8 | japonica | 12.5 | 12.7 |
|  | CS10CS2 | 1 | japonica | 20.2 | 20.4 |
|  |  |  | japonica | 41.4 | 41.7 |
|  |  | 6 | japonica | 5.6 | 5.6 |
|  |  | 8 | japonica | 5.8 | 5.8 |
| MRL | Ba10CS | 1 | japonica | 1 | 1 |
|  | Ab09CR | 12 | japonica | 27.3 | 27.3 |
|  | IR08CC | 1 | japonica | 36 | 36 |
|  |  | 3 | japonica | 16.5 | 16.5 |
|  |  | 4 | japonica | 32 | 32 |
|  |  | 6 | japonica | 2.9 | 2.9 |
|  |  |  | japonica | 3.1 | 3.1 |
|  |  |  | japonica | 9.1 | 9.1 |
|  |  |  | japonica | 23 | 23 |
|  |  | 7 | japonica | 1.1 | 1.1 |
|  |  |  | japonica | 2.1 | 2.1 |
|  |  |  | japonica | 5.1 | 5.1 |
|  |  | 8 | japonica | 1.1 | 1.1 |
|  |  |  | japonica | 4.1 | 4.1 |
|  |  | 10 | japonica | 4.1 | 4.1 |
|  |  | 11 | japonica | 0.1 | 0.1 |
|  |  | 12 | japonica | 0.2 | 0.2 |
| %MRL inc | IR08C | 6 | japonica | 4.6 | 4.6 |
| RDW | Ba10CC | 1 | japonica | 39.7 | 39.7 |
|  |  | 2 | japonica | 2 | 2 |
|  |  |  | japonica | 3.1 | 3.1 |
|  |  | 4 | japonica | 0.1 | 0.1 |
|  |  | 6 | japonica | 7.3 | 7.3 |
|  |  |  | japonica | 23 | 23 |
|  |  | 8 | japonica | 5.1 | 5.1 |
|  | Ba10CS | 1 | japonica | 39.7 | 39.7 |
|  |  | 6 | japonica | 4.1 | 4.1 |
|  |  | 12 | japonica | 24.8 | 24.8 |
|  | IR08FLS | 1 | japonica | 18.7 | 18.7 |
|  |  |  | japonica | 29.8 | 29.8 |
|  |  |  | japonica | 42.4 | 42.4 |
|  |  | 2 | japonica | 18.9 | 18.9 |
|  |  |  | japonica | 35.2 | 35.2 |
|  |  | 6 | japonica | 13.2 | 13.2 |
|  |  | 7 | japonica | 29.5 | 29.5 |
|  |  | 12 | japonica | 14.6 | 14.6 |
|  | IR08CC | 1 | japonica | 40.2 | 40.2 |
|  |  |  | japonica | 40.7 | 40.7 |
|  |  | 2 | japonica | 2 | 2 |
|  |  | 4 | japonica | 16.9 | 16.9 |
|  |  | 5 | japonica | 5 | 5 |
|  |  |  | japonica | 9.1 | 9.1 |
|  |  |  | japonica | 13.2 | 13.2 |
|  |  |  | japonica | 17.4 | 17.4 |
|  |  | 6 | japonica | 4.1 | 4.1 |
|  |  |  | japonica | 23.9 | 23.9 |
|  |  | 8 | japonica | 2.1 | 2.1 |
|  |  |  | japonica | 12.5 | 12.5 |
|  |  |  | japonica | 15 | 15 |
|  |  | 12 | japonica | 1.1 | 1.1 |
|  | IR08CS | 1 | japonica | 8.6 | 8.6 |
|  |  |  | japonica | 34.6 | 34.6 |
|  |  |  | japonica | 41.3 | 41.3 |
|  |  | 4 | japonica | 14.7 | 14.7 |
|  |  | 5 | japonica | 17.4 | 17.4 |
|  |  |  | japonica | 18 | 18 |
|  |  |  | japonica | 29.2 | 29.2 |
|  |  | 8 | japonica | 1.2 | 1.2 |
|  |  |  | japonica | 23.7 | 23.7 |
|  |  | 12 | japonica | 24.8 | 24.8 |
|  | IC09CC | 1 | japonica | 0.1 | 0.1 |
|  |  |  | japonica | 1 | 1 |
|  |  |  | japonica | 1 | 1 |
|  |  |  | japonica | 1 | 1 |
|  |  |  | japonica | 7.4 | 7.4 |
|  |  |  | japonica | 1 | 1 |
|  |  |  | japonica | 9.1 | 9.1 |
|  |  |  | japonica | 12.2 | 12.2 |
|  |  |  | japonica | 21.7 | 21.7 |
|  |  |  | japonica | 27.7 | 27.7 |
| yield | IR09wFLC | 1 | japonica | 3.2 | 3.2 |
|  |  |  | japonica | 23.6 | 25.4 |
|  |  |  | japonica | 26.8 | 26.8 |
|  |  |  | japonica | 29.8 | 29.8 |
|  |  |  | japonica | 31.9 | 32.1 |
|  |  |  | japonica | 42.6 | 43.6 |
|  |  | 2 | japonica | 22.2 | 22.2 |
|  |  |  | japonica | 35.2 | 35.2 |
|  |  | 3 | japonica | 12.7 | 12.7 |
|  |  |  | japonica | 26.1 | 26.3 |
|  |  | 4 | japonica | 2.3 | 2.5 |
|  |  |  | japonica | 20.8 | 20.8 |
|  |  | 5 | japonica | 28.6 | 28.7 |
|  |  | 7 | japonica | 19.3 | 19.7 |
|  |  |  | japonica | 29.5 | 29.7 |
|  |  | 9 | japonica | 0.9 | 1 |
|  |  | 10 | japonica | 5.7 | 6.1 |
|  |  | 11 | japonica | 17 | 17.7 |
|  |  |  | japonica | 20.8 | 21.3 |
|  |  |  | japonica | 22.9 | 23.1 |
|  |  | 12 | japonica | 9.7 | 10.2 |
|  | IR09wFLS | 1 | japonica | 3.2 | 3.8 |
|  |  |  | japonica | 23.6 | 32.1 |
|  |  |  | japonica | 42.6 | 43.6 |
|  |  | 2 | japonica | 0.7 | 0.8 |
|  |  |  | japonica | 10.5 | 11.1 |
|  |  |  | japonica | 22.2 | 23 |
|  |  |  | japonica | 24.4 | 24.8 |
|  |  |  | japonica | 34.9 | 35.2 |
|  |  | 3 | japonica | 12.4 | 12.7 |
|  |  |  | japonica | 16.1 | 16.4 |
|  |  |  | japonica | 26.1 | 27.6 |
|  |  | 4 | japonica | 2.3 | 2.5 |
|  |  |  | japonica | 30.5 | 30.7 |
|  |  |  | japonica | 32.6 | 33 |
|  |  | 5 | japonica | 18.9 | 19 |
|  |  |  | japonica | 28.6 | 28.7 |
|  |  | 6 | japonica | 26.9 | 27.2 |
|  |  | 7 | japonica | 19.3 | 19.7 |
|  |  |  | japonica | 22.2 | 22.3 |
|  |  |  | japonica | 26.5 | 29.7 |
|  |  | 8 | japonica | 5 | 5.3 |
|  |  |  | japonica | 21.3 | 21.6 |
|  |  | 9 | japonica | 0.9 | 1 |
|  |  |  | japonica | 10.4 | 12.3 |
|  |  | 10 | japonica | 5.7 | 6.1 |
|  |  |  | japonica | 12.8 | 13 |
|  |  |  | japonica | 19.8 | 19.8 |
|  |  | 11 | japonica | 5.8 | 7.1 |
|  |  |  | japonica | 16.7 | 17.7 |
|  |  |  | japonica | 19.3 | 21.3 |
|  |  | 12 | japonica | 2.7 | 3.4 |
|  |  |  | japonica | 9.7 | 10.2 |
|  |  |  | japonica | 15.4 | 15.9 |
|  | IR10FLC | 1 | japonica | 26.8 | 27.6 |
|  |  | 2 | japonica | 24.4 | 24.5 |
|  |  | 6 | japonica | 10 | 10.7 |
|  |  | 11 | japonica | 8.1 | 9.1 |
|  |  | 12 | japonica | 15.4 | 15.9 |
|  | IR10FLS | 1 | japonica | 19.7 | 19.7 |
|  |  |  | japonica | 24.1 | 25.4 |
|  |  |  | japonica | 26.8 | 26.8 |
|  |  |  | japonica | 42.6 | 42.8 |
|  |  | 2 | japonica | 10.4 | 11.1 |
|  |  |  | japonica | 22.2 | 22.2 |
|  |  | 4 | japonica | 2.3 | 2.5 |
|  |  |  | japonica | 33.9 | 34.4 |
|  |  | 5 | japonica | 23.4 | 23.9 |
|  |  |  | japonica | 28.6 | 28.7 |
|  |  | 8 | japonica | 21.4 | 21.6 |
|  |  | 11 | japonica | 17 | 17.3 |
|  |  |  | japonica | 20.8 | 21.3 |
|  | IR12FLS | 8 | japonica | 21.6 | 21.6 |
|  | IR12FLC | 1 | japonica | 26.9 | 26.9 |
|  |  | 2 | japonica | 24.4 | 24.5 |
|  |  | 8 | japonica | 21.6 | 21.6 |
|  | Af12FC | 1 | japonica | 40.2 | 40.2 |
