# Supplementary material for: Environmental Response and Genomic Regions Correlated with Rice Root Growth and Yield under Drought in the OryzaSNP Panel across Multiple Study Systems
Source: PLoS One. 2015 Apr 24;10(4):e0124127. doi: 10.1371/journal.pone.0124127 (PMC4409324; doi:10.1371/journal.pone.0124127)
Supplement: S1 Fig — (DOCX) [file pone.0124127.s012.docx]

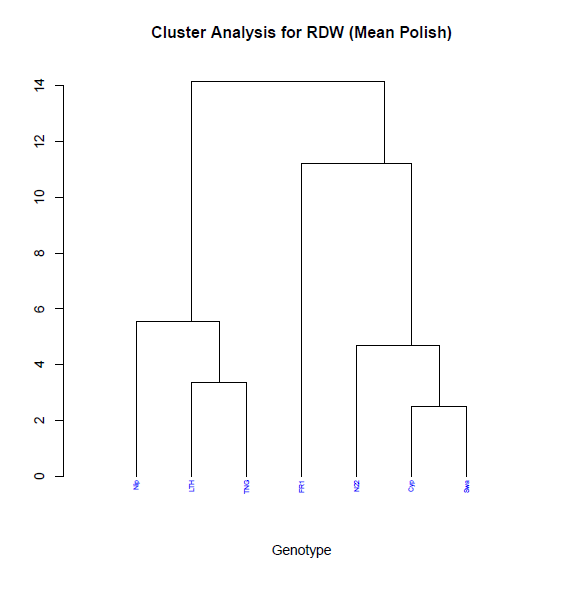

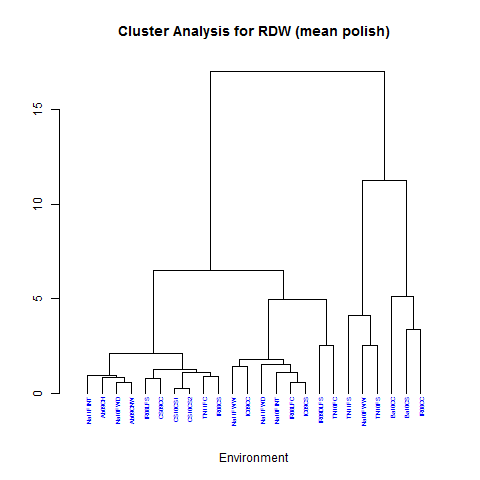

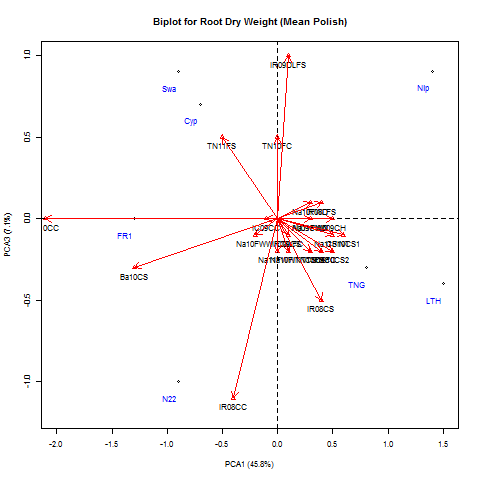

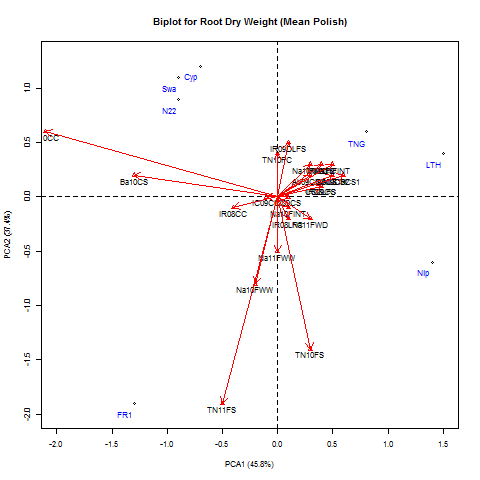


**S1 Fig. 1**. Analysis including the subset of 7 OryzaSNP genotypes phenotyped for RDW in the line-source system at Nagoya Univ.
